# Neural network-based interface reconstruction algorithm for two-phase fluid flow

**DOI:** 10.1016/j.fmre.2025.01.018

**Published:** 2025-02-15

**Authors:** Junhua Gong, Yujie Chen, Bo Yu, Dongliang Sun, Bohong Wang, Guoyun Shi, Bin Chen

**Affiliations:** 1 State Key Laboratory of Multiphase Flow in Power Engineering, Xi'an Jiaotong University, Xi'an 710049, China; 2 School of Mechanical Engineering, Beijing Institute of Petrochemical Technology, Beijing 102617, China; 3 School of Petroleum Engineering, Yangtze University, Wuhan 430100, China; 4 National & Local Joint Engineering Research Center of Harbor Oil & Gas Storage and Transportation Technology/Zhejiang Key Laboratory of Pollution Control for Port-Petrochemical Industry, Zhejiang Ocean University, Zhoushan 316022, China; 5 Kunlun Digital Intelligence Technology Company, Beijing 102266, China

**Keywords:** Curve reconstruction, Neural network, Two-phase flow, ANN, CNN

## Abstract

In two-phase fluid flow, the vapor-liquid interface tends to behave as a curved shape under the influence of surface tension. Curve reconstruction favors improving the resolution of bubbles or droplets in numerical studies. Based on artificial neural network (ANN) and convolutional neural network (CNN), two curve reconstruction algorithms, namely CIR-ANN and CIR-CNN, are proposed in this study. Both algorithms can achieve high-precision prediction of the center and radius when reconstructing interfaces using a portion of a standard circle, especially for the CIR-CNN algorithm. A strict mass conservation strategy is also proposed to ensure the reliability of the neural network predictions. In comparison with interface reconstruction algorithms such as piecewise linear interface construction (PLIC) algorithm, efficient least squares volume-of-fluid interface reconstruction algorithm (ELVIRA), quadratic spline based interface (QUASI) reconstruction algorithm after the first interface correction, Circle-based Interface Reconstruction (CIR), and CIR-ANN algorithm, the proposed CIR-CNN demonstrates good advantages in static interface reconstruction, with average accuracy ratios of 48.40, 86.69, 26.34, 4.07 and 2.26. However, when capturing the bubble under a complex rotation and shear recovery flow field, the advantage of the proposed algorithms decreases due to the increased complexity of the fluid volume fraction distributions. Regarding the computational time cost of reconstructing random circular interfaces, the proposed algorithms achieve average reduction ratios of 4.16, 7.86, and 34.14, respectively, compared to the CIR, ELVIRA, and QUASI algorithms.

## Graphical abstract

**Figure:**
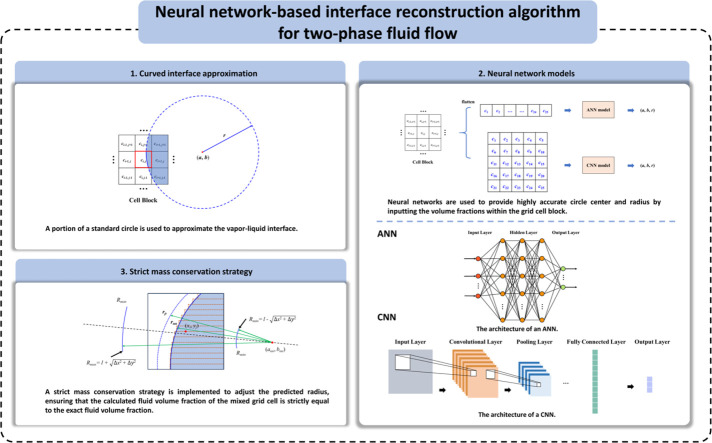

## 1 Introduction

Multiphase flow phenomena are common in both natural and industrial fields, such as ocean waves [1] and clouds [2] in nature, oil-gas separation in the petroleum industry [3], the flow of water in geothermal wells [4], bubbles in water electrolysis for hydrogen production [[5], [6], [7]], and metal melting in the metallurgical industry [8]. Numerical multiphase flow simulation can help deepen the understanding of the underlying principles behind these complex flow phenomena, which is significant in scientific research and engineering practice [9]. In numerical simulations of non-miscible fluid multiphase flow, determining the position of the vapor-liquid interface is crucial. There are two main methods for determining the position of the interface: interface tracking and interface capturing. Interface tracking methods explicitly track the position of the interface during the flow process, such as the Front Tracking method [10]. Interface capturing methods obtain the interface position implicitly by solving the phase function, such as the Volume of fluid (VOF) method [11] and the Level Set method [12]. The VOF method utilizes the volume fraction to represent the proportion of volume occupied by the main phase fluid within the grid cell. This method has an excellent property of mass conservation, one of the key factors contributing to its widespread adoption [13].

The geometric methods offer high accuracy in solving the VOF function. One of the most commonly used geometric solving methods is the piecewise linear interface construction (PLIC) algorithm [14], which uses line segment and plane to approximate the vapor-liquid interface within each mixed grid cell for two-dimensional and three-dimensional problems. In the PLIC methods, the normal vector or slope of the interface and the constant term associated with the interface position collectively determine the final position of the reconstructed interface. The constant term is usually determined by the mass conservation constraint, and precisely calculating the normal vector of the reconstructed interface becomes the study focus.

Puckett and Saltzman [15] calculated the centroid coordinates by averaging all products of volume fraction and center coordinates of grid cells within a 3 × 3 grid cell block. Subsequently, they determined the interface normal vector based on the centroid position and the center of the central grid cell. In contrast to the method of Puckett and Saltzman, Parker and Youngs [16] performed a weighted average of all products of volume fraction and center coordinate of grid cells within a 3 × 3 grid cell block. Grid cells located on the diagonal in the 3 × 3 grid cell block were assigned a weight of 2 in the calculation process, while the rest of the grid cells were assigned a weight of 1. Scardovelli and Zaleski [17] first calculated the normal vectors at the four vertices of each grid cell within a 3 × 3 grid cell block, then averaged these normal vectors to obtain the final normal vector at the center of the central grid cell. Pilliod and Puckett [18] calculated the total volume fractions of the grid cells in the left and right columns within a 3 × 3 grid cell block. They then used half of the difference between these two column volume fraction totals as the slope of the interface.

To further enhance the accuracy of the PLIC algorithm, scholars have proposed a series of methods such as the least squares volume-of-fluid interface reconstruction algorithm (LVIRA) [19], efficient least squares VOF interface reconstruction algorithm (ELVIRA) [20], the linear least-square fit method [17] and spline-based interface reconstruction algorithm (SIR) [21]. The LVIRA algorithm determined the normal vector of the linear interface by minimizing the error function related to the volume fraction. The ELVIRA method calculated six slopes based on different difference formats and selected the slope with the minimum error as the slope of the linear interface. The linear least-square fit method first determined the "radius of influence" zone and then applied it to the reference points within it to fit and determine the slope of the line segment representing the interface. The SIR algorithm [21] first used the PLIC algorithm to reconstruct the initial interface. Then, the center points of those line segments were collected, and cubic spline interpolation through those points was used to adjust the orientations of those linear segments.

Neural networks have become an alternative to numerical methods for solving problems. Compared to traditional numerical methods, well-trained neural networks can quickly provide accurate results with input data, simplifying the computation process. This characteristic helps reduce the implementational difficulty and improve computational efficiency for the PLIC algorithm in the two-phase fluid flow problem. Therefore, neural networks have recently been utilized to determine the normal vector (or slope) and constant of the linear reconstruction interface using the PLIC algorithm.

Svyetlichnyy [22] utilized a simple neural network to predict the normal vector of the interface based on the volume fractions of 8 grid cells. The neural network did not directly provide the specific values of the normal vector. Instead, it outputted the sine value of the angle associated with the normal vector. Svyetlichnyy then applied the well-trained neural network to reconstruct test cases for interfaces with diverse shapes. The results of the tests showed that the neural network-based method for calculating the normal vector had smaller errors compared to numerical methods, such as the center of mass method and the central difference method. Li et al. [23] extended the neural network-based approach for predicting interface normal vector into the three-dimensional problem. They employed the volume fractions of the target grid cell and its 26 neighboring grid cells, along with curvature from the target grid cell, as inputs to the neural network. Unlike the model proposed by Svyetlichnyy [22], their model directly outputted the component values of the normal vector. However, only two component values and the sign of the third component of the normal vector were outputted, necessitating subsequent normalization to determine the exact value of the third component. Andrew Cahaly et al. [24] adopted a method similar to Li et al. [23] for predicting the normal vector of the interface. They replaced the curvature in Li et al.'s neural network input with two phasic barycenters at each grid cell within a 3 × 3 × 3 grid cell block. Their neural network subsequently directly outputted all three component values of the normal vector. To address the issues of high computational cost and low accuracy in reconstructing interfaces using the VOF method on unstructured grids, Nakano et al. [25] utilized three GNN models to predict the parameters of the reconstructed interfaces. By inputting the coordinates of the vertices of each tetrahedral cell and the volume fraction of the cell, the trained models outputted the normal vector, curvature, center, and area of the reconstructed interface for each tetrahedral cell. Regarding the constant term in the interface equation, Ataei et al. [26] employed a multilayer perceptron (a type of simple feedforward neural network) to predict the constant term of the interface equation. They input the angle *θ* between the interface normal vector and the *x*-coordinate axis, and the volume fraction *α*_0_ into the neural network in two-dimensional problems. In three-dimensional problems, they input the polar angle *θ*, azimuth angle *φ*, and the volume fraction *α*_0_ into the neural network.

The vapor-liquid interface tends to form a curved shape due to the influence of surface tension [27,28]. Therefore, those interface reconstruction algorithms using the linear representation may not be accurate enough. Many scholars have successively proposed curve reconstruction algorithms to improve interface reconstruction accuracy.

Chorin[29] utilized a portion of an osculating circle to approximate the interface inside a mixed grid cell, which is determined by three parameters. By continuously adjusting these three parameters through trial and error, the circle was adjusted to intersect with the 3 × 3 grid cell block to give the same volume fraction of each grid cell within the grid cell block. Scardovelli and Zaleski [17] used a linear interface reconstruction algorithm to reconstruct the initial interface and then determined the radius influence zone based on the initial interface. The midpoints and endpoints of the linear interface inside the radius influence zone served as reference points, and the quadratic least-square fitting method was used to determine the parameters in the general form equation of the circular interface. Diwakar et al. [30] similarly employed the PLIC algorithm to obtain the initial interface, then made the first correction to the interface, transforming the initial discontinuous linear interface segments to continuous piecewise parabolic curves. Subsequently, based on the mass conservation constraint, the interface underwent the second correction to ensure the final interface possessed both continuity and smoothness. Chen et al. [27,28] proposed a horizontal refined piecewise curve reconstruction (HOPCIR) algorithm and a circle-based interface reconstruction (CIR) algorithm with the characteristics of straightforward implementation and high accuracy. Both algorithms employed a portion of a standard circle to approximate the vapor-liquid interface within a grid cell. The radius of the standard circle was calculated using the interface curvature and signed distance. The center coordinates of the circle were determined through the dichotomy method and were subject to the mass conservation constraint. These curve reconstruction algorithms significantly enhance computational accuracy. For example, the CIR algorithm improves the accuracy of reconstructing circular interfaces by an average of 17.35 times compared to the PLIC algorithm.

The curve reconstruction algorithms above can improve reconstruction accuracy, but they often require complex implementation procedures, leading to increased computational costs. Chorin's method [29] involves a complex trial-and-error process to determine circular interface-related parameters. Sometimes, it may not yield circular interfaces that meet the specified requirements. Some [17,30] also require a prior linear reconstruction process to obtain additional information before proceeding with curve reconstruction. The principles of the algorithms proposed by Chen et al. [27,28] for curve reconstruction are relatively simple. However, the algorithms require the volume fractions and signed distances of the grid cells to calculate the radius and center coordinates. Computing the signed distance involves finding the shortest distance from the grid cell center to the reconstructed interface, which requires multiple iterative computation processes. The complex implementation procedure may hinder the utilization of those algorithms for efficient interface reconstruction. Neural networks can help reduce the implementation complexity for interface reconstruction and improve computational efficiency. However, current research [[22], [23], [24], [25], [26]] on neural network-based interface reconstruction mainly focuses on calculating normal vectors or constant terms for reconstructed linear interfaces and has not been extensively applied to complex and accurate curve reconstruction.

To this end, an accurate, straightforward, and efficient method called the neural network-based curve interface reconstruction (CIR-NN) algorithm is proposed in this paper for reconstructing the vapor-liquid interface within the two-dimensional framework. The CIR-NN algorithm uses a portion of a standard circle to approximate the vapor-liquid interface within the mixed grid cell, and the neural network is used to provide accurate high-precision circle center coordinates and radius. The implementation procedure for reconstructing the interface of the CIR-NN algorithm will be comprehensively introduced first. Furthermore, the accuracy of the CIR-NN algorithm will be evaluated through a comprehensive comparison with other algorithms. The time cost for the interface reconstruction using the CIR-NN algorithm is also discussed. The CIR-NN algorithm aims to provide an accurate, straightforward, and efficient method for reconstructing curved vapor-liquid interfaces.

## 2 Idea and implementation procedure of the CIR-NN algorithm

### 2.1 Curve interface reconstruction using a portion of a circle

In the two-dimensional framework, the vapor-liquid interface tends to behave as a circular shape under the influence of surface tension. Therefore, a portion of a circle is mainly used by many scholars to approximate the vapor-liquid interface in mixed grid cells.

In Chorin's method [29], the vapor-liquid interface was approximated by a portion of a circle described by parameters *ρ, θ,* and *r*. The *ρ* represents the distance from the origin of the polar coordinate system to the center of the circle, *θ* represents the angle measured counterclockwise from the positive direction of the polar axis to the vector from the origin of the coordinate system to the center of the circle, and the *r* is the radius of the circle. Then, the specific values of those parameters were determined using the complex trial-and-error method. Scardovelli et al. [17] used a portion of a circle with a general-form equation to approximate the interface, and the equation had three parameters to be determined: d, *e*, and *f*. These parameters were determined by applying a quadratic least-square fit to the reference points. It is necessary first to perform linear interface reconstruction, then determine the radius influence zone, and finally select the midpoint or endpoint of the linear interface located within the radius influence zone as the reference point. Chen et al. [27,28] approximated the vapor-liquid interface using a portion of a standard circle, which was described by center coordinates (*a, b*) and radius *r*. The radius was determined first based on the curvature and sign function, and then the specific center coordinates of the circle were determined based on the mass conservation constraint. However, the calculation process involves the sign distance function, necessitating a complex calculation procedure.

**Fig. 1:**
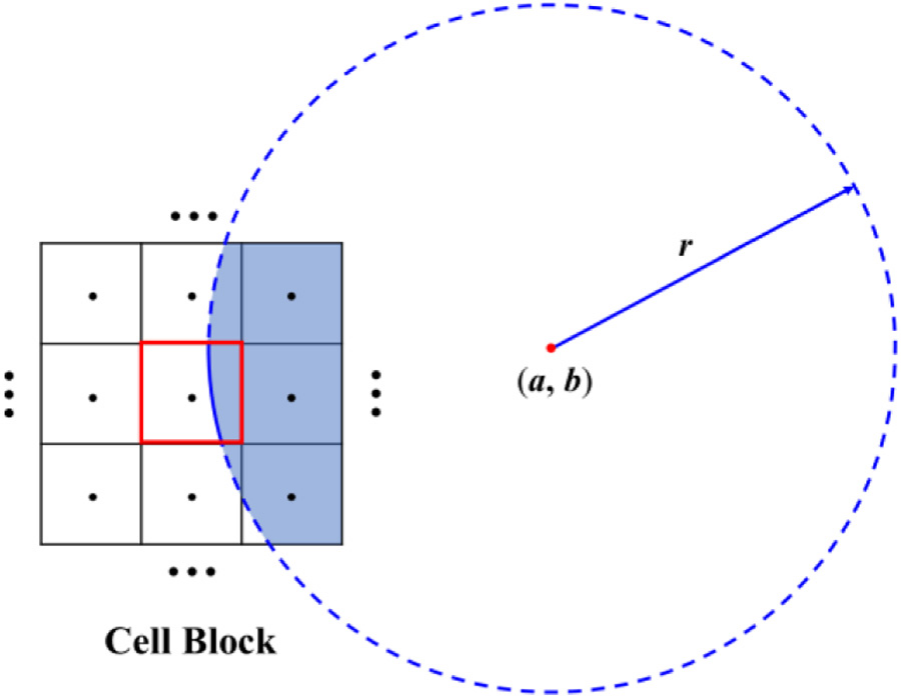
**Approximation of the vapor-liquid interface using a portion of a standard circle**.

Each of the scholars above has proposed a curve reconstruction algorithm that uses a portion of a circle to approximate the vapor-liquid interface. The main differences among these algorithms lie in how the circle is represented and the methods used to determine the related parameters. The process of determining the parameters of the circle in these algorithms is relatively complex, which may hinder the efficiency of the algorithm. To overcome this problem, neural networks can be utilized to quickly determine those circle-related parameters, thereby improving the efficiency of interface reconstruction. Among these circle representations of those algorithms, the one proposed by Chen et al. [27,28] is simpler and more straightforward, thus facilitating integration with neural networks for interface reconstruction. In the algorithm proposed by Chen et al., a portion of a circle described by Eq. (1) is adopted for curve reconstruction, as shown in Fig. 1. Therefore, neural networks are employed to directly predict the center coordinates and radius of the circular interface with high accuracy in this paper.

(1) $${\left(x-a\right)}^{2}+{\left(y-b\right)}^{2}=r^{2}$$

where *a* and *b* represent the *x* and *y* coordinates of the circle center, respectively, and *r* represents the radius of the circle.

### 2.2 Neural network models construction

#### 2.2.1 Basics of neural networks

When using the neural network for curve interface reconstruction, it is essential first to determine its input, output, and specific type of neural network. As mentioned earlier, the proposed CIR-NN algorithm also uses a portion of a circle to approximate the vapor-liquid interface, and the neural network is employed to provide the center coordinates and radius of the circle quickly. Consequently, the network output is determined as the *x*-coordinate *a* and *y*-coordinate *b* of the center of the reconstructed circular interface, along with the radius *r* of the circular interface. In previous studies on interface reconstruction employing neural networks, the volume fractions of all grid cells within a grid cell block were commonly input into the neural network. This is primarily because the volume fraction is an easily obtainable and crucial parameter within the VOF framework. Therefore, utilizing volume fraction as the input of the neural network is both reasonable and appropriate. Based on this consideration, the input to the neural network in this paper is determined as all the volume fractions inside the grid cell block. The shape of the grid cell block used in this paper is 5 × 5.

**Fig. 2:**
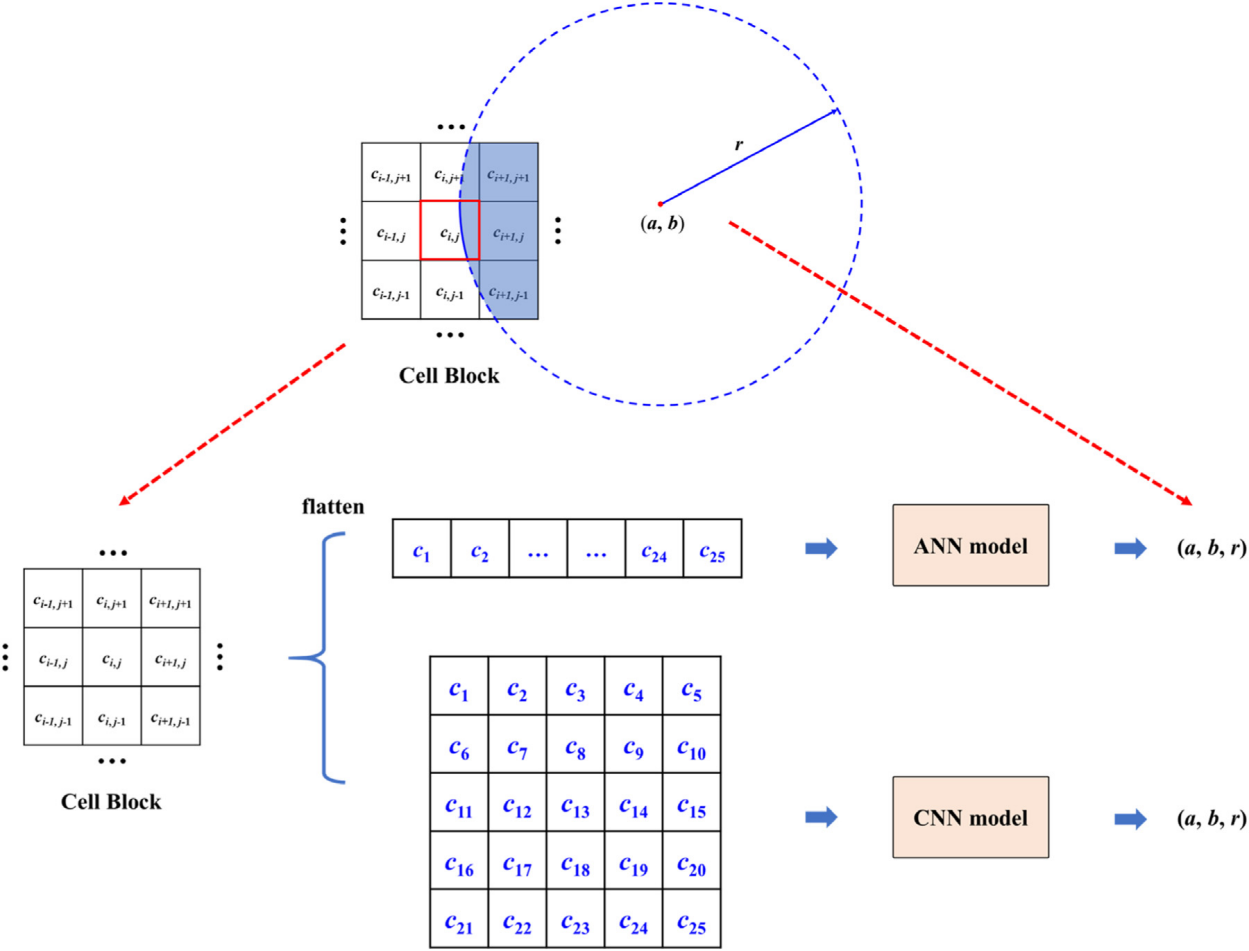
**Input, output, and the specific type of neural networks used in the paper**.

Artificial Neural Network (ANN) [31] is one of the most commonly used neural networks and has shown better performance in many studies [[21], [22], [23]]. Therefore, ANN is a preferable choice. Considering the spatial relationship between volume fractions of grid cells, the Convolutional Neural Network (CNN) [32] demonstrates better effectiveness in handling such data. Hence, CNN is the alternative neural network adopted in this paper. It is important to note that ANN and CNN have different formatting requirements for input. The volume fractions of the 5 × 5 grid cell block cannot be directly input into the ANN, and they need to be flattened into one-dimensional data to meet the data requirement of the ANN. In contrast, the CNN can directly process volume fractions with a shape of 5 × 5 without requiring an additional flattening process, which enables the input volume fractions of the CNN model to preserve the original spatial relationship. The finalized input, output, and the specific type of neural networks selected are shown in Fig. 2.

**Fig. 3:**
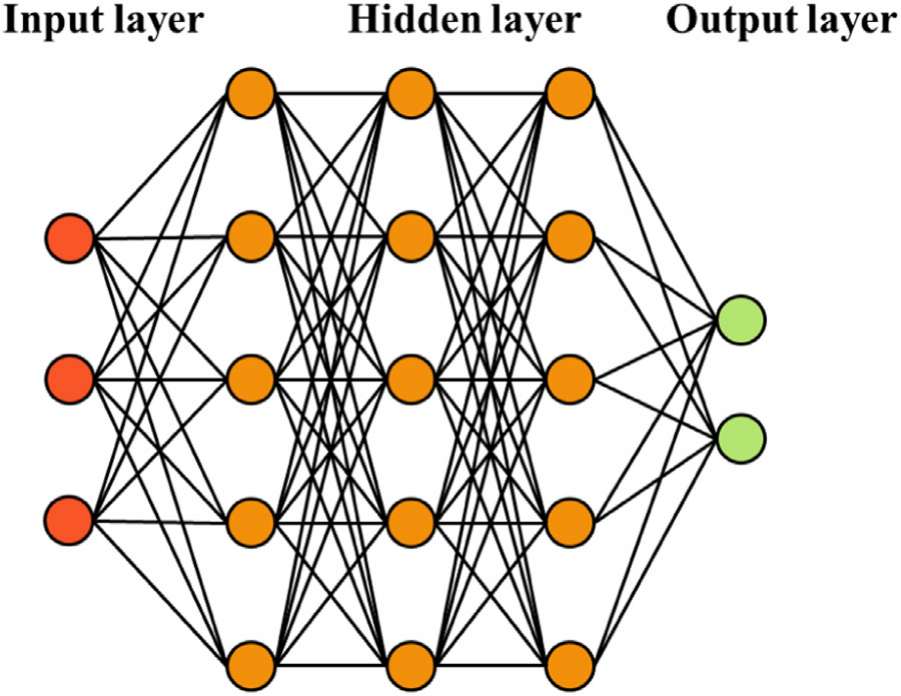
**General architecture of ANN**.

**Fig. 4:**
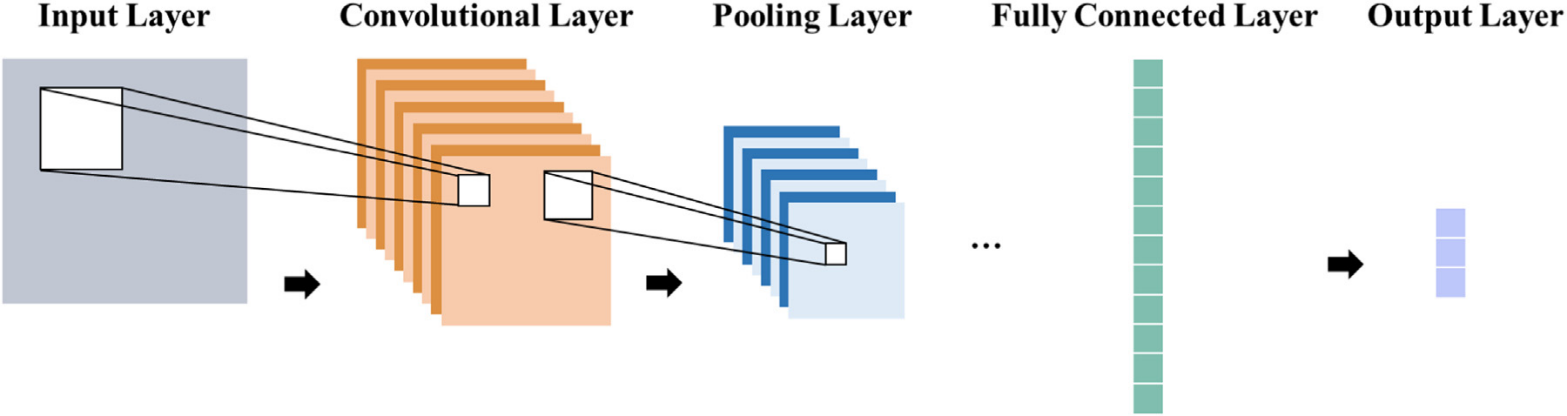
**General architecture of CNN**.

As shown in Fig. 3, an ANN mainly consists of an input layer, hidden layers with varying numbers of neurons in each layer, and an output layer. The ANN primarily extracts information from the data within these hidden layers. The specific number of hidden layers and neurons in each hidden layer can be adjusted based on the particular problem. As shown in Fig. 4, A CNN generally consists of an input layer, convolutional layers, pooling layers, fully connected layers, and an output layer. The convolutional layers, the essence of the CNN [33], are highly efficient at extracting information from data. The CNN structure mentioned above is the general structure, and an appropriate architecture can also be chosen as needed for specific problems. The neural network structures are relatively basic, and their performance can be enhanced by optimizing hyperparameters and determining the best loss function.

**Fig. 5:**
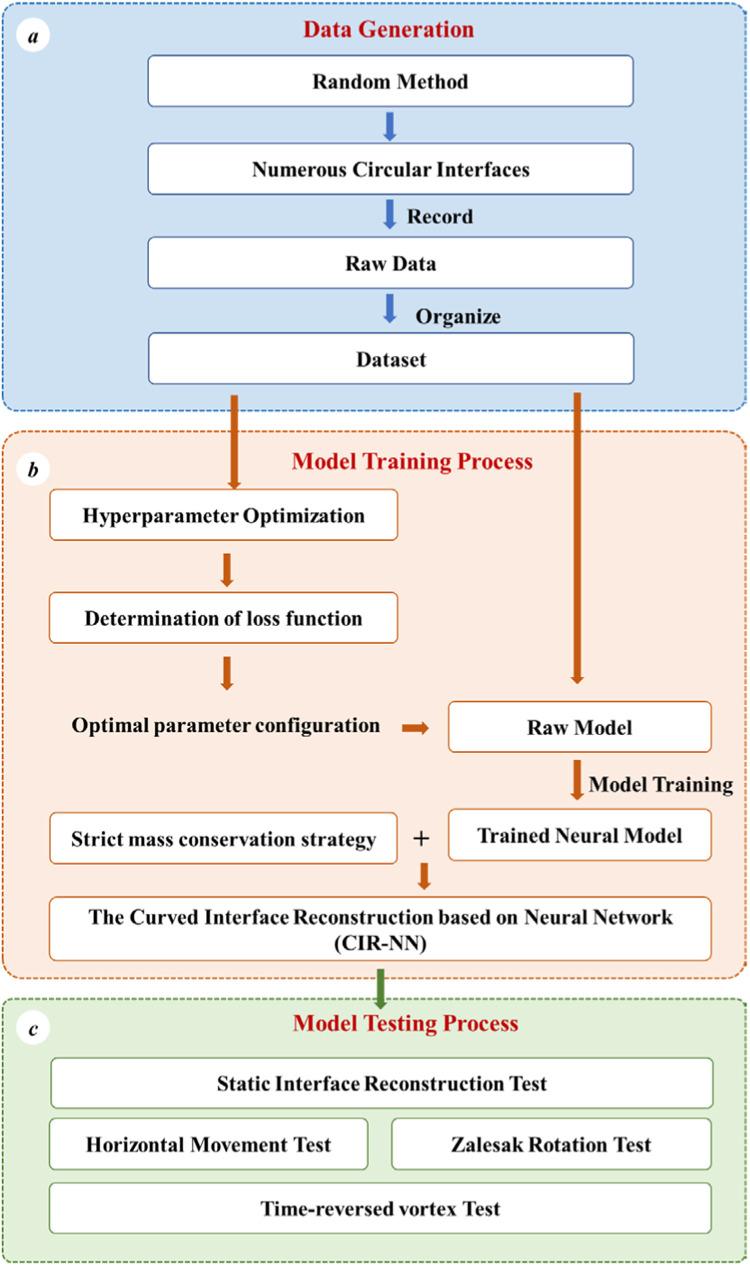
**Construction, testing, and application processes of the curved interface reconstruction algorithm based on neural networks**.

The construction, testing, and application processes of the curved interface reconstruction algorithm based on neural networks are illustrated in Fig. 5.

#### 2.2.2 Data generation

A random method is employed to create numerous circles within the computational domain to generate data for neural network training. Initially, different point coordinates and line segment lengths are randomly generated within the computational domain to serve as the centers and radii of the interfaces. Based on the intersection of circles with grid cells, the grid cells within the computational domain can be categorized into three types: exterior grid cells (entirely outside the circle), interior grid cells (entirely inside the circle), and mixed grid cells (intersecting with the circle). The volume fraction of an exterior grid cell is set to 0 (or 1 if the main phase fluid is gas), and the volume fraction for an interior grid cell is set to 1 (or 0 if the primary phase fluid is gas). The volume fraction of a mixed grid cell is precisely determined by dividing the mixed grid cell into many small rectangular pieces, calculating the volume fraction in each piece, and finally summing these volume fractions of small pieces. The volume fractions of each grid cell block centered on the mixed grid cell are recorded along with the corresponding circle center coordinates and radius. The recorded volume fractions of a grid cell block serve as the input data for neural networks, while the output data of these neural networks are the recorded circle center coordinates and radius.

**Fig. 6:**
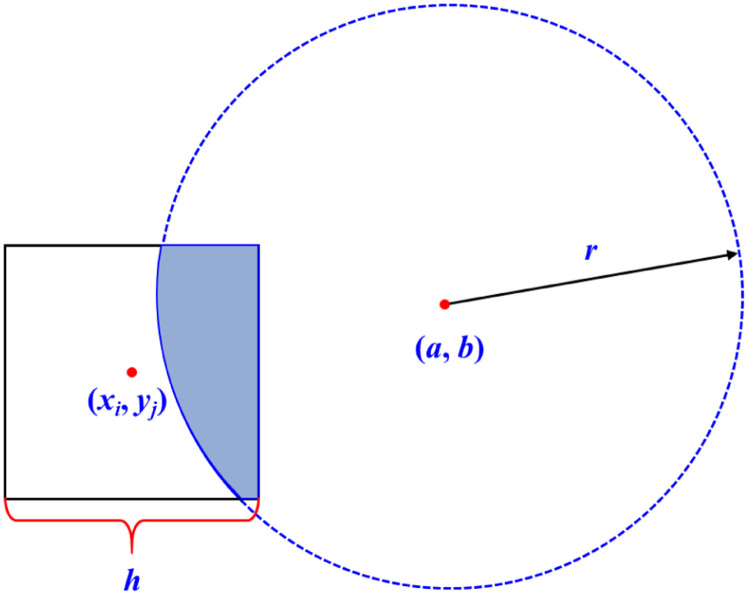
**Intersection between the approximated circular interface and the mixed grid cell**.

However, the recorded volume fractions, circle center coordinates, and radius constitute raw data that cannot be directly used for model training. As depicted in Fig. 6, the raw data exhibits several issues. Firstly, the recorded circle center coordinates are in the absolute coordinate system. Due to the random method used to generate the circle center coordinates, the generated center coordinates are distributed across the entire computational domain. This results in a wide range of variations in the recorded center coordinates, which may not be conducive to training the neural network. When applying neural networks for prediction, using a relative coordinate system with the center of the grid cell block as the origin to represent the circle center coordinates is also more convenient. Secondly, the grid spacing step is constant within the computational domain have fixed widths, hindering the ability of the well-trained neural network model to reconstruct the curved interface under different grid spacing steps. To address these issues, the center coordinate is transformed into relative coordinates with the origin at the center of the grid cell block. Concurrently, the center coordinates and radius are normalized by dividing them by the grid spacing step, yielding data independent of the grid spacing step. The specific process is illustrated in Eqs. (2) ∼ (4).

(2) $$a^{\prime }=\left(a-x_{i}\right)/h$$

(3) $$b^{\prime }=\left(b-y_{j}\right)/h$$

(4) $$r^{\prime }=r/h$$

#### 2.2.3 Hyperparameter optimization

**Fig. 7:**
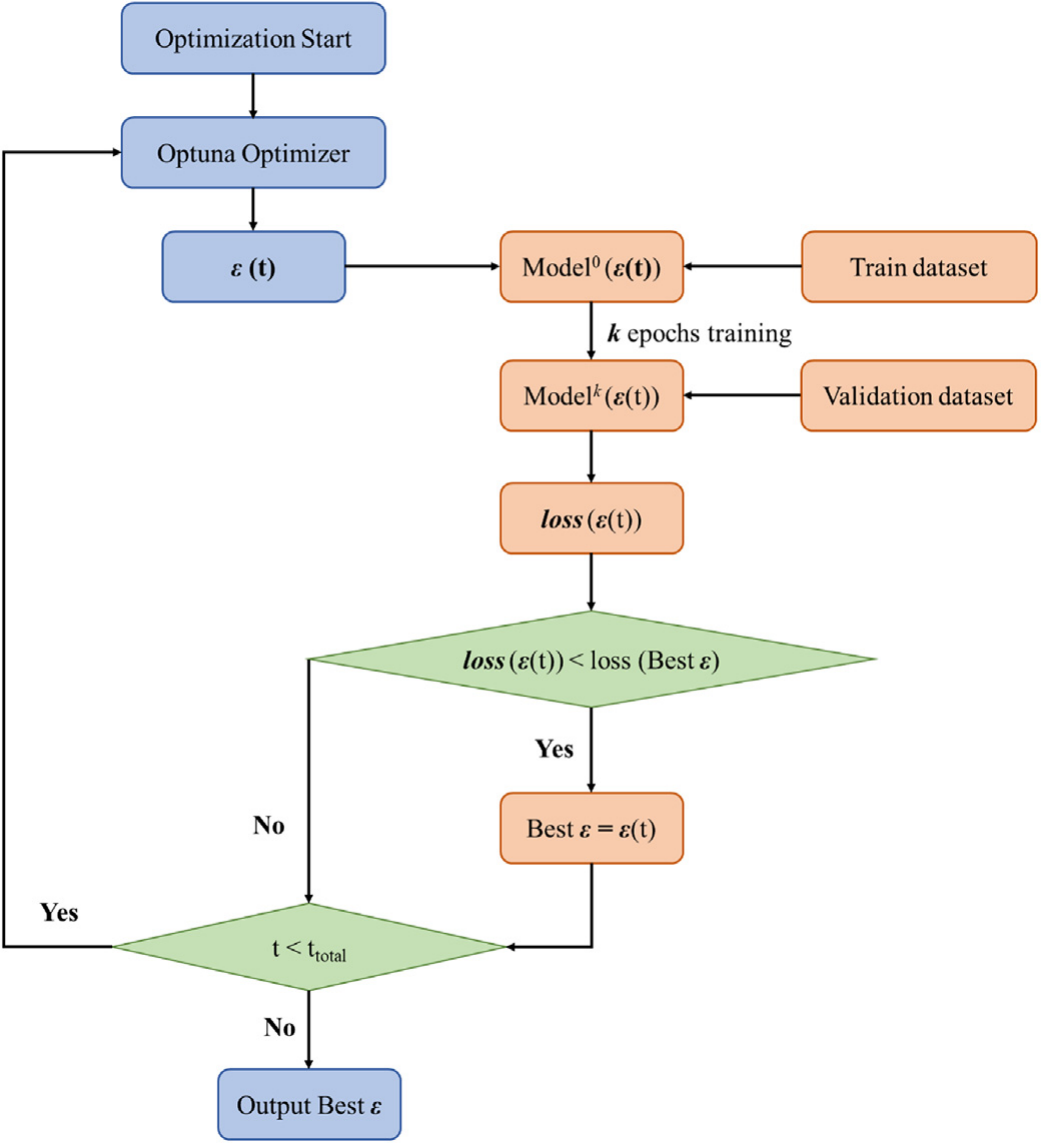
**Process of hyperparameter optimization using the Optuna optimizer**.

Optimizing the hyperparameters of neural networks can effectively enhance their performance and improve prediction accuracy. Common hyperparameter optimization methods include grid search [34], random search [35], and Bayesian optimization [36], and those incorporate heuristic algorithms [[37], [38], [39]]. Compared to other algorithms, Bayesian optimization is a probabilistic method that can adjust optimization parameters using prior information and demonstrates superior optimization performance [40,41]. Optuna, an efficient hyperparameter tuning framework that can achieve the Bayesian optimization [42], is chosen in this study to optimize the hyperparameters of the proposed neural networks. The optimization process using the Optuna optimizer is illustrated in Fig. 7.

In Fig. 7, *ε*(*t*) represents the values of the hyperparameters optimized by the Optuna during the *t*-th optimization trial. Model^0^(ε(t)) denotes the raw neural network model that utilizes the hyperparameters values *ε*(*t*), with the superscript 0 indicating that the model has not undergone any training yet. As the model goes through *k* (6000) training epochs, Model*^k^*(*ε*(*t*)) emerges, representing the trained neural network model. Loss(*ε*(*t*)) acts as a metric for evaluating the performance of Model^k^(*ε*(*t*)) on the validation dataset, with a lower value indicating better model performance. Best *ε* refers to the recorded optimal hyperparameter combination, and the entire hyperparameter optimization process aims to identify the hyperparameter values that result in the lowest loss value on the validation dataset. Finally, *t*_total_ denotes the total number of optimization trials set for the optimization process.

**Fig. 8:**
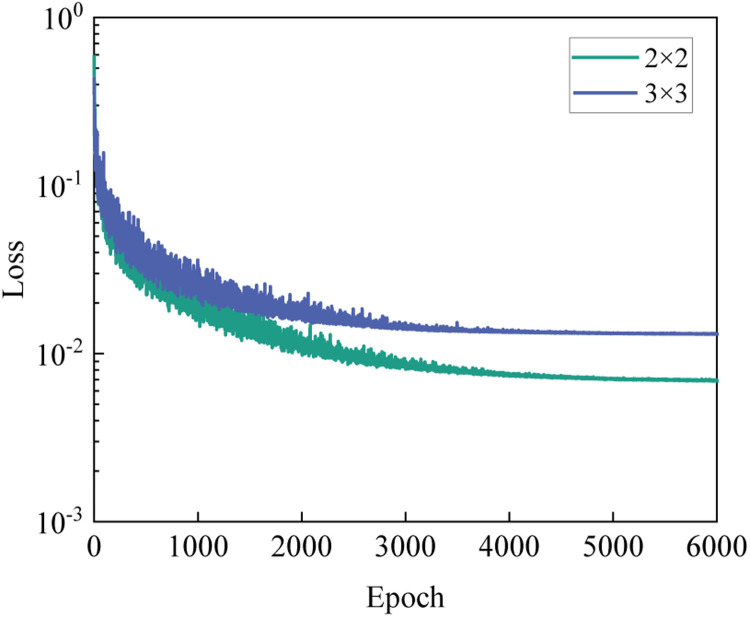
**Evolution of relative losses over epochs of the CNN model on the validation dataset under different convolutional kernel sizes**.

Before optimizing the hyperparameters of a CNN, it is crucial to determine the size of the convolutional kernel. Although the kernel size can be considered a hyperparameter to be optimized alongside other hyperparameters using the Optuna optimizer, this approach may lead to ineffective optimization trials due to the significant impact of kernel size on the CNN structure and performance. An effective strategy to minimize unnecessary optimization trials is identifying a suitable kernel size before comprehensively optimizing the remaining hyperparameters. This study has two options for the convolutional kernel size in the convolutional layer: 2 × 2 and 3 × 3. While using different kernel sizes may result in varying output data shapes within the last convolutional layer, this shape can be made consistent through appropriate adjustments, ensuring that the subsequent calculation process of the fully connected layer remains unaffected. The evolution of relative losses over epochs of the CNN model on the validation dataset under different convolutional kernel sizes is illustrated in Fig. 8. The results reveal that the CNN model using a 2 × 2 convolutional kernel demonstrates a lower loss during the training process compared to its counterpart with a 3 × 3 convolutional kernel. This finding may stem from the fact that smaller kernels can capture local features of the data with greater precision. It is worth noting, however, that smaller kernels necessitate stacking more convolutional layers to process data with the same size. Based on the observation, in the subsequent hyperparameter optimization process, the CNN will default to adopting the 2 × 2 convolutional kernel and further refine other hyperparameters. It is noted that, as shown in Fig. 4, the general architecture of CNN includes pooling layers. However, considering that the shape of the grid cell (5 × 5) is relatively small, incorporating a pooling layer would significantly reduce the spatial resolution of the feature maps, thereby limiting the effectiveness of feature extraction. To address this issue, pooling layers were omitted in the architecture of the CNN adopted in this paper, allowing for a greater number of convolutional layers to enhance feature extraction.

For ANN, the hyperparameters include the initial learning rate, the number of hidden layers, and the number of neurons in each hidden layer. As for CNN, the size of the convolutional kernel is already determined, and the number of convolutional layers is indirectly determined. Except for the initial learning rate, the hyperparameters include the number of fully connected layers and the number of neurons in each fully connected layer. Both the hidden layer of ANN and the fully connected layer of CNN are linear layers. The hyperparameters need to be optimized, and their ranges are listed in Table 1.

**Table 1:** **Hyperparameters of CNN and ANN models**.

Model	Optimized hyperparameter	Range
ANN	Learning rate	0.1–0.01
	Number of the hidden layer	2–6
	Number of neurons	15–35
CNN	Learning rate	0.1–0.01
	Number of the fully connected layer	1–2
	Number of neurons	15–35

In the optimization process of two neural networks, the learning rate gradually decreases as the training epochs increase. The formula for calculating the learning rate at different training epochs is illustrated in Eq. (5).

(5) $$\eta \left(t\right)=\eta_{0}\cdot \gamma^{t//d}$$

where *η* represents the learning rate for the current training epoch; *η*_0_ is the initial learning rate; *γ* is the decay rate of the learning rate, 0.925; *t* stands for the current training epoch; d is the decay step of the learning rate, indicating that the learning rate decays once every d epochs. In this paper, the parameter d is set to 100.

**Fig. 9:**
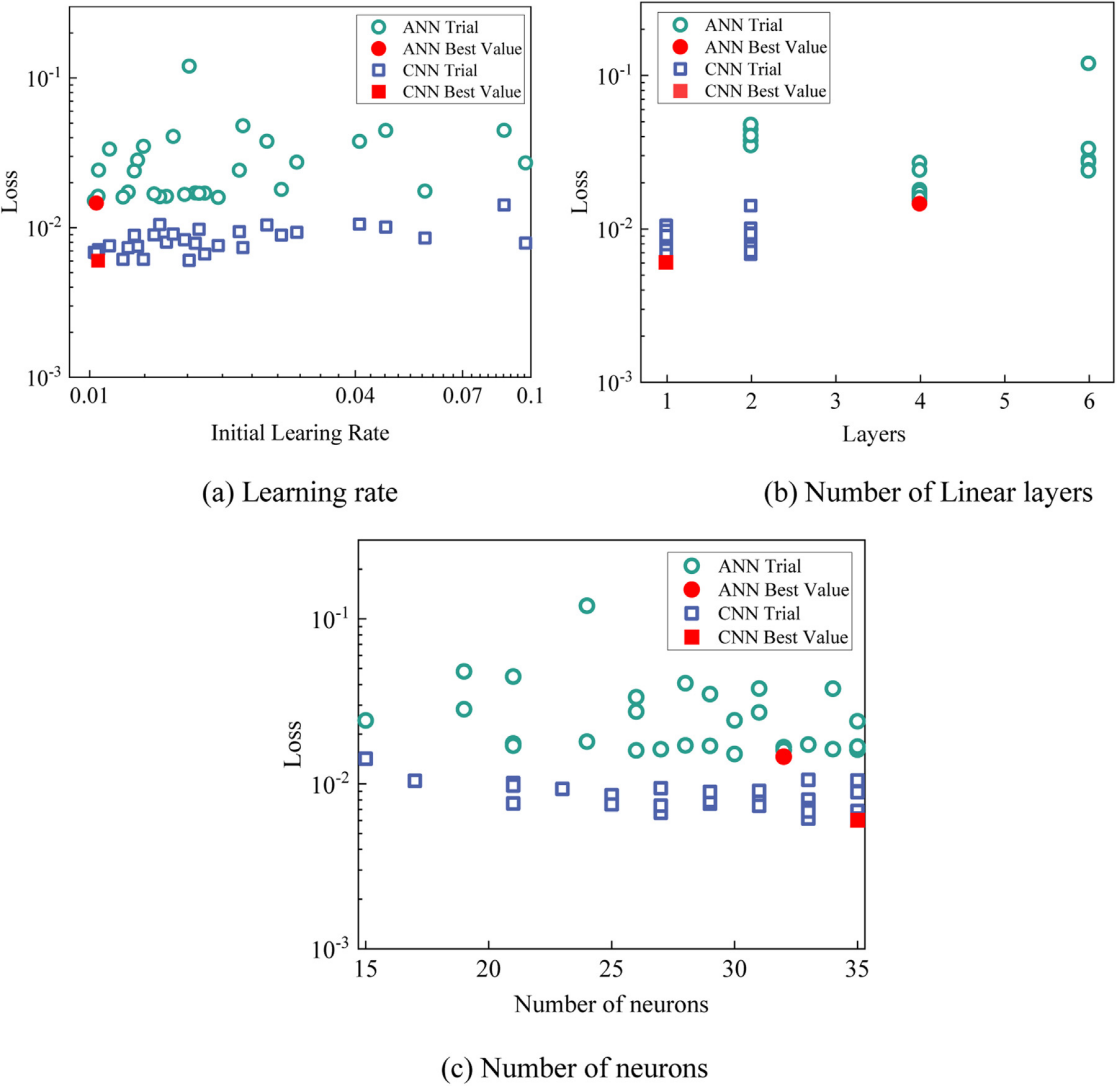
**Variation of (a) learning rate, (b) number of linear layers, and (c) number of neurons during the hyperparameter optimization process**.

Thirty rounds of independent hyperparameter optimization processes are conducted for each ANN and CNN in this study. During these optimization processes, the variations of the losses on the validation dataset resulting from changes in different hyperparameter values are illustrated in Fig. 9. Under different hyperparameter conditions, different losses exhibit clusters and sparse regions. This is because the Optuna optimizer can roughly identify the zone where the optimal hyperparameter values lie as the number of optimization trials increases. Consequently, the loss values of trials start to cluster gradually within this region, narrowing down the search range until the optimization process concludes. As shown in Fig. 9(a), it is evident that a larger initial learning rate is not always better, and the values of the initial learning rate of both ANN and CNN tend to cluster around smaller value zones. CNN primarily relies on convolutional layers to extract features from data, while fully connected (linear) layers aggregate and process these features to generate the final output. When the network includes several convolutional layers, the feature representation capability is typically sufficient. Consequently, the number of fully connected layers can be reduced to lower computational complexity and limit the total number of model parameters. To achieve a balance between model accuracy and computational efficiency, this study sets the optimization range for fully connected layers to 1 to 2 layers. The optimization results indicate that using a single fully connected layer in the CNN model yields better performance. It suggests that employing one linear layer to process the information after convolution is sufficient (Fig. 9(b)). ANN relies more on hidden layers for information extraction. Hence, the optimization results suggest a higher number of hidden layers are needed. Similarly, a higher number of neurons aids in better information extraction, leading to the optimization results favoring a higher number of neurons (Fig. 9(c)). After 30 rounds of hyperparameter optimization, the optimal values for different parameters of the two neural networks are presented in Table 2. The architectures of ANN and CNN are determined by determining the linear layers and the number of neurons within the linear layers of the two neural networks. Detailed information about the finalized ANN and CNN architectures can be found in Appendix A.

**Table 2:** **Best values of hyperparameters**.

Model	Optimized hyperparameter	Best value
ANN	Learning rate	0.014189671
	Number of the hidden layer	4
	Number of neurons	32
CNN	Learning rate	0.010447231
	Number of the fully connected layer	1
	Number of neurons	35

#### 2.2.4 Loss function embedded the mass conservation constraint

Conventionally, the relative error function used for training neural networks is outlined as follows in Eq. (6).

(6) $$E_{1}=\frac{1}{n}\sum_{i=1}^{n}\frac{\sqrt{{\left({\bar{a}}_{i}-a_{i}\right)}^{2}+{\left({\bar{b}}_{i}-b_{i}\right)}^{2}+{\left({\bar{r}}_{i}-r_{i}\right)}^{2}}}{\sqrt{a_{i}^{2}+b_{i}^{2}+r_{i}^{2}}}$$

where $\bar{a}$ and $\bar{b}$ represent the predicted *x*-coordinate and *y*-coordinate of the circle center by neural network, respectively; $\bar{r}$ is the predicted radius of the circle by neural network; *a* and *b* is the exact *x*-coordinate and *y*-coordinated of the circle center, respectively; *r* is the exact radius of the circle.

The mass conservation is essential for the two-phase flow simulation. Therefore, the volume fraction of the main phase region enclosed by the predicted reconstructed interface and mixed cell faces should equal the exact volume fraction, ensuring the mass conservation constraint. To achieve this goal as much as possible, a loss term representing the degree of mass conservation is embedded into the original loss function, and the final loss function is listed in Eqs. (7) and (8).

(7) $$E_{2}=\frac{1}{n}\sum_{i=1}^{n}\frac{\sqrt{{\left({\bar{a}}_{i}-a_{i}\right)}^{2}+{\left({\bar{b}}_{i}-b_{i}\right)}^{2}+{\left({\bar{r}}_{i}-r_{i}\right)}^{2}+\omega {\left({\bar{c}}_{i}-c_{i}\right)}^{2}}}{\sqrt{a_{i}^{2}+b_{i}^{2}+r_{i}^{2}}}$$

(8) $${\bar{c}}_{i}=f\left({\bar{a}}_{i},{\bar{b}}_{i},{\bar{r}}_{i}\right)$$

where *c* represents the exact fluid volume fraction of the mixed grid cell; $\bar{c}$ represents the volume fraction of the mixed grid cell calculated based on predicted parameters ($\bar{a}$, $\bar{b}$ and $\bar{r}$) from the neural network; *ω* is the adjustment coefficient used to adjust the impact of mass conservation constraint and training data on model training performance; *f* is the function for calculating the volume fraction.

**Fig. 10:**
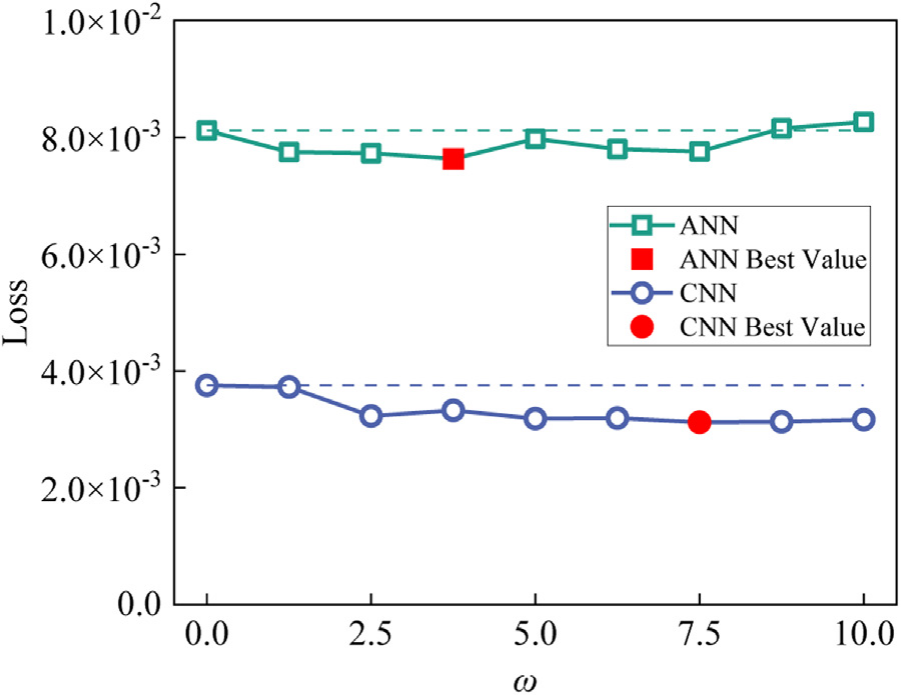
**Profiles of the losses vary *ω* for the ANN and CNN models**.

Adjusting the coefficient of this added loss term in the loss function can determine the optimal loss function with the best performance. Specifically, the range of the *ω* in this study is set as 0.0 to 10.0. Variations in the final loss of the models on the validation dataset under different ω values are presented in Fig. 10. With the *ω* value increases, the ANN and CNN models do not show a monotonic loss trend on the validation dataset. When *ω* is 3.75 and 7.5, the ANN and CNN models achieve the lowest loss, respectively. Appropriately embedding a term that represents the degree of mass conservation into the loss function can reduce the losses of ANN and CNN models on the validation dataset, thereby enhancing their performance.

**Fig. 11:**
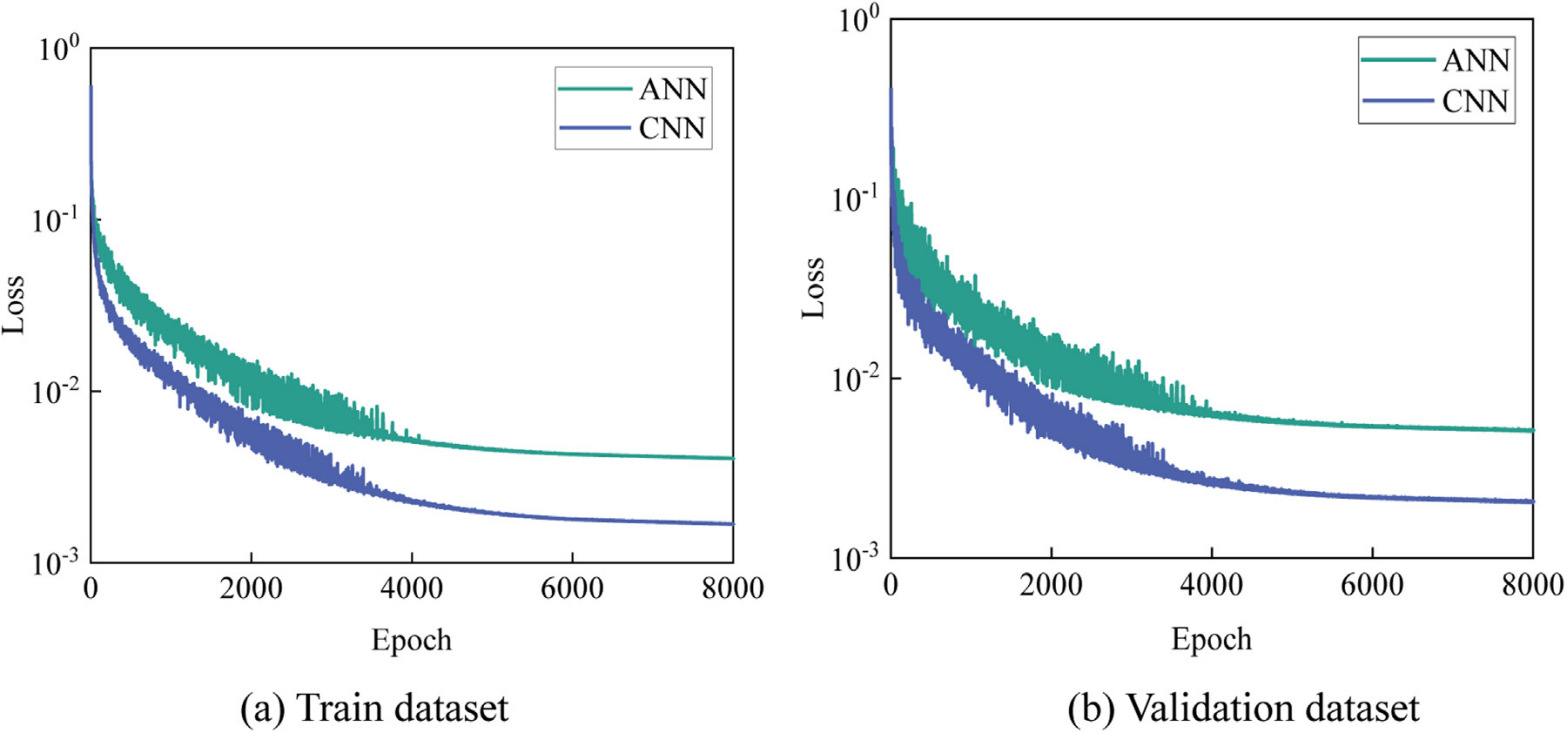
**Evolution of relative losses over epochs of the ANN model and CNN model trained with the final determined loss function on (a) train dataset and (b) validation dataset**.

After completing hyperparameter optimization and determining the specific form of the loss function, the optimal configuration is applied to both the ANN and CNN models. Under the optimal configuration, the evolution of relative losses over epochs of the two models on the complete training and testing datasets are shown in Fig. 11. After 8000 training epochs, the ANN model has a loss of 0.511% on the validation dataset, while the CNN model has a loss of 0.205%. The CNN model has a lower loss on the validation dataset than the ANN model, indicating that the trained CNN model performs better.

#### 2.2.5 Evaluation of the well-trained neural network models on the test dataset

During the previous training process, the neural network models showed low losses on both the train and validation datasets, indicating good performance. However, the performance of the well-trained neural network models has not yet been evaluated. This subsection will provide a comprehensive evaluation of the performance of the neural network models on the test dataset.

The Root Mean Square Error (RMSE) and Coefficient of Determination (R^2^) are commonly used to assess the performance of neural network models on test datasets. A smaller RMSE value and a larger R^2^ value indicate better performance of the neural network models. The calculation methods for RMSE and R^2^ are shown in Eqs. (9) and (10), respectively.

(9) $$RMSE=\sqrt{\frac{1}{n}\sum_{i=1}^{n}{\left(\phi_{i}-\phi_{i}^{P}\right)}^{2}}$$

(10) $$R^{2}=1-\frac{\sum_{i=1}^{n}{\left(\phi_{i}-\phi_{i}^{P}\right)}^{2}}{\sum_{i=1}^{n}{\left({\bar{\phi }}_{i}-\phi_{i}\right)}^{2}}$$

Where *n* represents the amount of data in the dataset; *ϕ* stands for the label value of the predicted variable; $\phi^{P}$ is the output value of the neural networks; $\bar{\phi }$ signifies the average of the exact values. The variables *ϕ* can represent the *x*-coordinate (*a*), *y*-coordinate (*b*), and radius (*r*) of the circle.

**Fig. 12:**
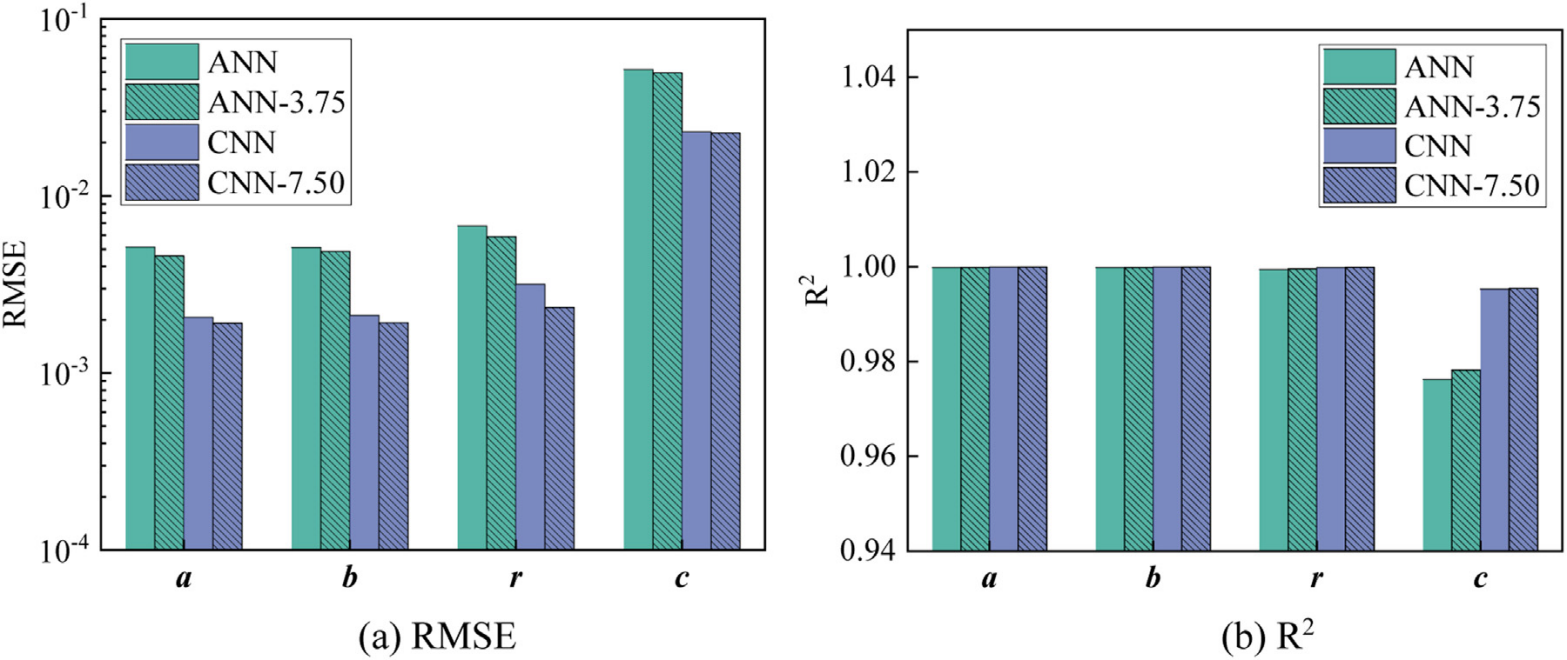
**Evaluation results include (a) RMSE and (b) R^2^ of the ANN and CNN models on the test dataset**.

As mentioned earlier, an additional loss term representing the degree of mass conservation has been incorporated into the original loss function. By carefully adjusting the coefficient of this loss term, it has been observed that both the ANN-3.75 and the CNN-7.50 models have achieved smaller loss values on the validation dataset than the ANN and CNN models training with the original loss function. The numbers following the model names represent the optimal coefficients of the embedded loss terms in the final determined loss function. The four models, namely ANN, ANN-3.75, CNN, and CNN-7.50, perform calculations on the test dataset. ANN and CNN models are trained with the original loss function after 8000 epochs. The ANN-3.75 and CNN-7.50 are the models with the loss function with the optimal values of the coefficient *ω* determined in the proceeding section after 8000 training epochs. The calculation results are evaluated using the RMSE and R^2^ metrics, and the evaluation results are shown in Fig. 12 and Table 3. The RMSE values of the predicted center coordinates and radii (after normalized) by the ANN and ANN-3.75 models are below 6.8 × 10^–3^, while for the CNN and CNN-7.50 models, their RMSE values are even lower, below 3.2 × 10^–3^. Additionally, the R^2^ values of the predicted center coordinates and radii from the four neural network models are very close to 1.0. Those quantitative results indicate that those neural network models exhibit good performance and provide highly accurate predictions. In comparing the prediction accuracy, the CNN models (CNN and CNN-7.50 models) exhibit lower RMSE values and higher R^2^ values than the ANN models (ANN and ANN-3.75 models), suggesting that the CNN models have higher prediction accuracy. This finding is consistent with the lower loss values observed during the training process of the CNN models. The superior performance of the CNN models can be primarily attributed to the significant spatial structural relationships within their training data and their proficiency in processing data that exhibit spatial structure. As mentioned in the previous section on data generation, it is necessary to record the volume fractions of the grid cells surrounding the labeled ones, which inherently possess strong spatial interdependencies. However, ANN models typically adopt the method of flattening the two-dimensional volume fraction data into one-dimensional vectors. This flattening process destroys the spatial relationships of the data, leading to poorer performance of the ANN models when dealing with spatially structured data [43]. In contrast, CNN models do not require additional data flattening, thus naturally preserving the spatial relationships. Furthermore, the convolution operation of CNNs is especially adept at processing data with spatial structure and can efficiently extract spatial features. Consequently, the CNN models significantly surpass the ANN models in their ability to process such data.

**Table 3:** **Evaluation of the ANN and CNN models using RMSE and R^2^ on the test dataset**.

	Model	*a*	*b*	*r*	*c*
RMSE	ANN	5.15 × 10^–3^	5.12 × 10^–2^	6.75 × 10^–3^	5.17 × 10^–2^
	ANN-3.75	4.58 × 10^–3^	4.86 × 10^–3^	5.89 × 10^–3^	4.95 × 10^–2^
	CNN	2.06 × 10^–3^	2.11 × 10^–3^	3.17 × 10^–3^	2.30 × 10^–2^
	CNN-7.50	1.92 × 10^–3^	1.92 × 10^–3^	2.35 × 10^–3^	2.26 × 10^–2^
R^2^	ANN	0.999845	0.999848	0.999418	0.976219
	ANN-3.75	0.999877	0.999864	0.999558	0.978198
	CNN	0.999975	0.999974	0.999872	0.995304
	CNN-7.50	0.999979	0.999979	0.999930	0.995464

As mentioned earlier, an additional loss term representing the degree of mass conservation has been incorporated into the original loss function. By carefully adjusting the coefficient of this loss term, it has been observed that the ANN-3.75 and the CNN-7.50 models have achieved smaller loss values on the validation dataset than their original ANN and CNN models. As depicted in Fig. 12, both the ANN-3.75 model and the CNN-7.50 model exhibit smaller RMSE values and higher R^2^ values than ANN and CNN models when predicting the coordinates of the circle center and its radius. This result aligns with the reduction in loss values of the ANN-3.75 and CNN-7.50 models on the validation dataset, providing additional confirmation of the enhancement in model performance by appropriately adding the loss term representing the degree of mass conservation. It is worth noting that although the performance of the ANN-3.75 and CNN-7.50 models has improved due to the inclusion of the additional physical constraint loss term, the CNN-7.50 model still outperforms the ANN-3.75, which is attributed to the inherent advantage of the CNN model in handling data with spatial relationships. In the subsequent section, unless otherwise specified, the ANN model specifically refers to the ANN-3.75 model, while the CNN model specifically refers to the CNN-7.50 model.

As shown in Table 3, compared to the direct predictions of the center coordinates and radii produced by the neural network, the volume fractions calculated based on these predicted parameters exhibit a larger RMSE value and a smaller R^2^ value, which means poorer performance. This is primarily because, despite the high accuracy in predicting the center coordinates and radii, there are inevitably slight errors in the exact values. These errors will collectively affect the calculation process of volume fractions. As a result, the discrepancy between the calculated volume fraction and the exact volume fraction is larger than the discrepancies observed for the predicted circle center coordinates and radii. This error of calculated volume fraction will accumulate during the bubble or droplet evolution, leading to unphysical dynamic behaviors.

#### 2.2.6 Strict mass conservation strategy

As previously mentioned, the parameters predicted by neural networks are highly accurate. However, despite this level of accuracy, the volume fraction calculated using these parameters consistently exhibits minor discrepancy from the exact value, resulting in a non-strict fulfillment of the mass conservation constraint. This discrepancy will accumulate during the bubble or droplet evolution, leading to unphysical dynamic behaviors. A strict mass conservation strategy is proposed in this study to address this discrepancy.

**Fig. 13:**
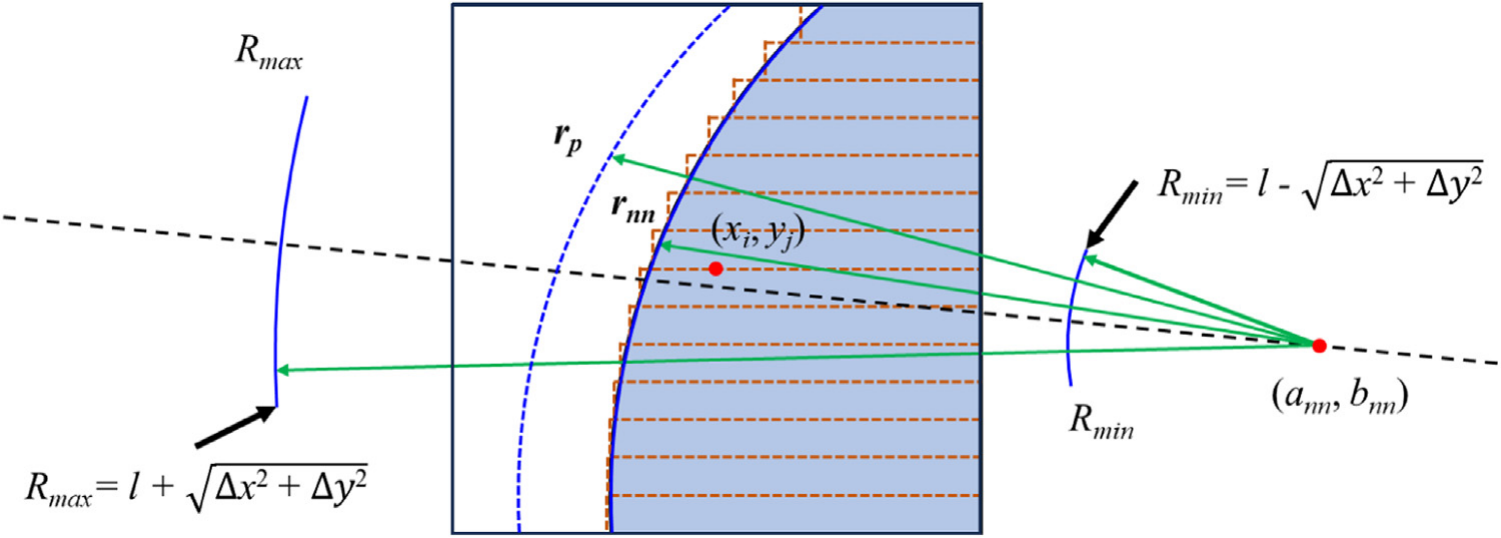
**Sketch of strictly mass conservation strategy**.

As shown in Fig. 13, this strategy first calculates the maximum value *R*_max_ and minimum value *R*_min_ of the possible radius changes during the correction process, using Eqs. (11)∼(13), based on the circle center coordinates (*a_nn_, b_nn_*) predicted by the neural network models, the center coordinates of the grid cell (*x_i_, y_j_*), and the width of the grid cell (Δ*x* and Δ*y*). Subsequently, an iterative method is employed within the calculated range (*R*_min_, *R*_max_) to determine the radius *r_p_*, which can ensure the calculated volume fraction of the mixed grid cell is strictly equal to the exact volume fraction. After implementing the strict mass conservation strategy, the radius of the approximated circle of the interface has changed from the *r_nn_* predicted by the neural network models to *r_p_*.

**Fig. 14:**
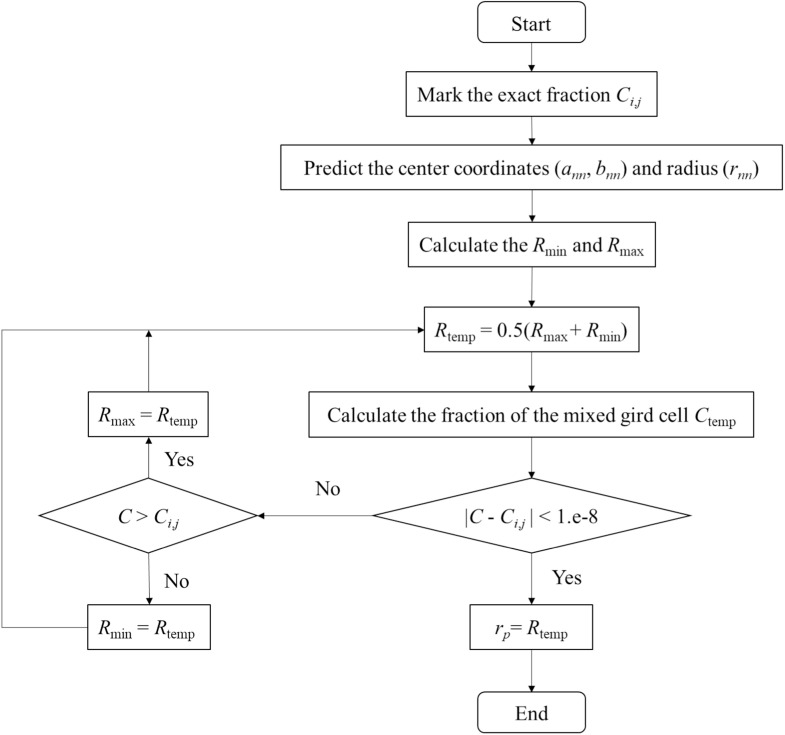
**Determining the radius strictly satisfying mass conservation constraint via dichotomy**.

Using the dichotomy method as an example, the process of determining the radius *r_p_* is detailed in Fig. 14. Firstly, calculate the maximum *R*_max_ and minimum *R*_min_ values within the potential range of radius changes. Take the midpoint value of the potential range of radius changes as the intermediate radius variable *R*_temp_. Then, based on this *R*_temp_, along with the existing coordinates of the circle center (*a_nn_, b_nn_*), calculate the volume fraction *C*_temp_ of the portion enclosed by the circular interface and the mixed grid cell. As shown in Fig. 13, the volume fraction calculation uses a relatively simple method. It divides the part of the mixed grid cell occupied by the main phase fluid into many small pieces of fixed width, calculates the volume fraction of the main phase fluid within each piece, and sums these volume fractions to obtain the total volume fraction of the mixed grid cell. The specific calculation process can be found in the references [24,25]. Next, determine if the calculated fraction *C*_temp_ meets the accuracy requirement compared to the exact volume fraction C_i,_*_j_*. If the current result *R*_temp_ does not meet the requirement, adjust the range of the radius changes based on the current *R*_temp_ and repeat the above calculation process until the accuracy requirement is met, ultimately obtaining *r_p_*.

(11) $$l=\sqrt{{\left(a_{nn}-x_{i}\right)}^{2}+{\left(b_{nn}-y_{j}\right)}^{2}}$$

(12) $$R_{\min}=l-\sqrt{\Delta x^{2}+\Delta y^{2}}$$

(13) $$R_{\max}=l+\sqrt{\Delta x^{2}}+\Delta y^{2}$$

**Fig. 15:**
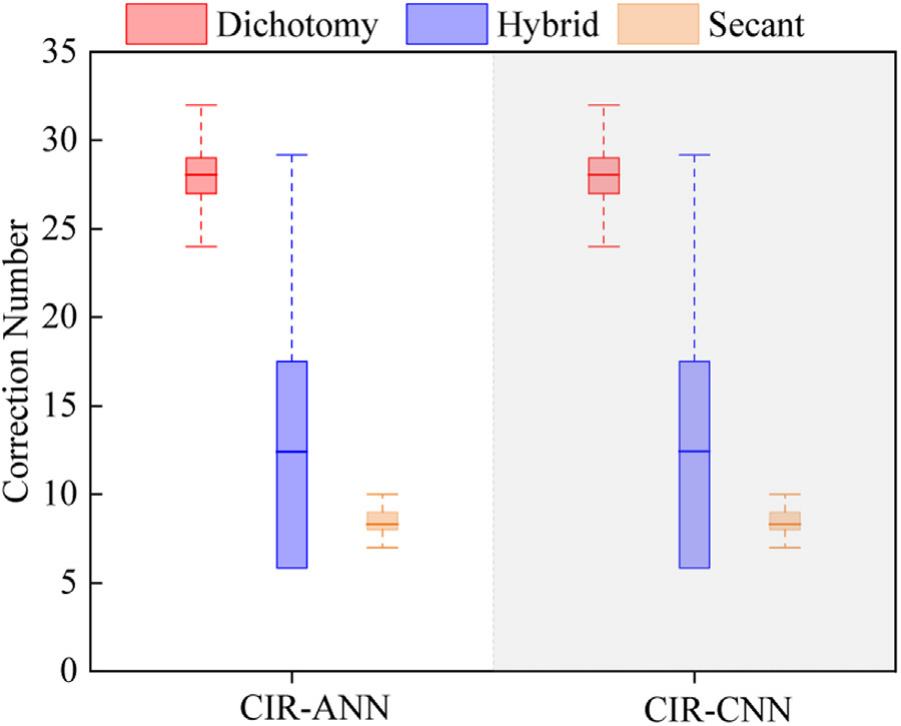
**Boxplot of the number of corrections during the correction process**.

The distribution of the number of corrections applied to the predicted radius from the neural network using the dichotomy is shown in Fig. 15. The distributions of corrections for both the CIR-ANN and CIR-CNN algorithms are similar, with an average of 28 correction numbers for each algorithm. Although there is no significant difference in the number of corrections between the two algorithms, it is noteworthy that, as mentioned previously, the CNN model of the CIR-CNN algorithm demonstrates higher accuracy in predicting the center coordinates of the circle compared to the ANN model of the CIR-ANN algorithm. Consequently, the interface reconstructed by the CIR-CNN algorithm is more precise than that of the CIR-ANN algorithm. This will be explained further in the subsequent section of the static interface reconstruction test.

The correction process within the strict mass conservation strategy necessitates multiple invocations of the volume fraction calculation function. An excessive number of corrections (averaging 28 for the dichotomy) can lead to a substantial increase in the time consumption of the strategy, thereby adversely reducing the efficiency of the CIR-ANN and CIR-CNN algorithms. Consequently, adopting a more efficient correction method within the strict mass conservation strategy is crucial to minimize the number of corrections and increase the overall efficiency of the CIR-ANN and CIR-CNN algorithms.

**Fig. 16:**
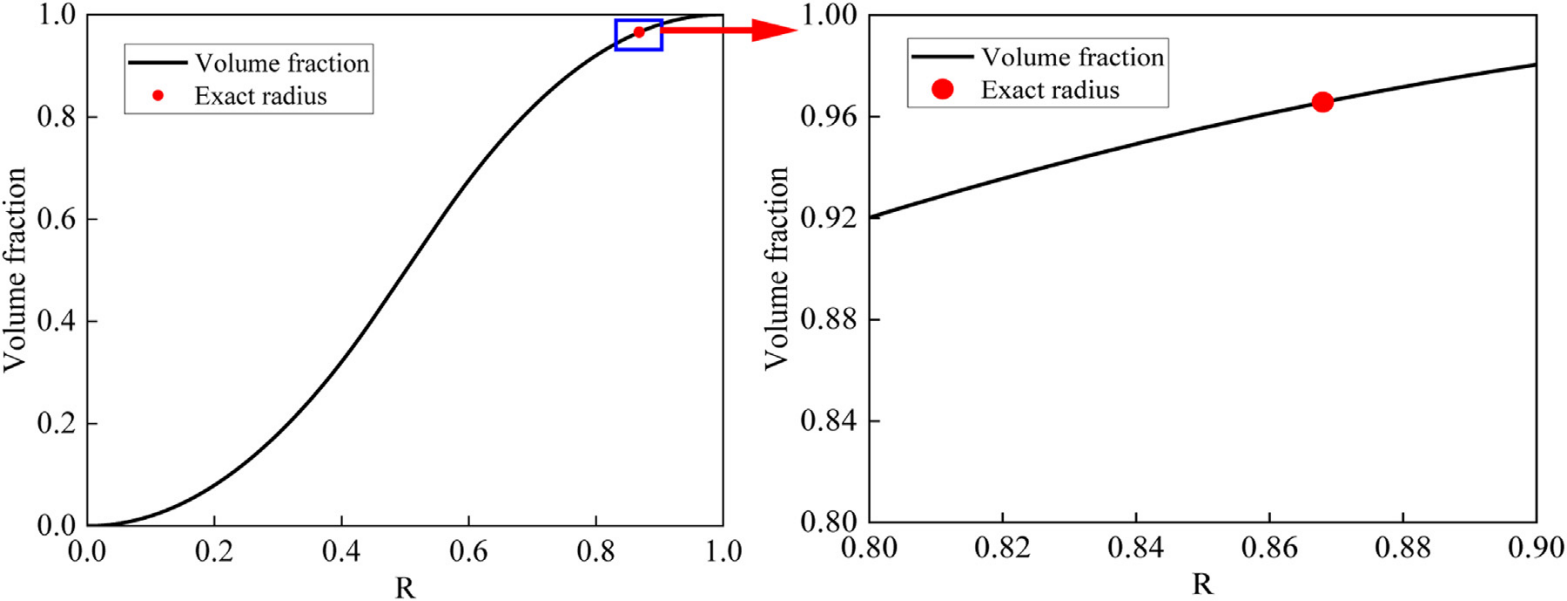
**Volume fraction of mixed grid cell for different radii**.

In the strict mass conservation strategy, the method of calculating the temporary radius using the dichotomy is shown in Eq. (14). The inefficiency of the dichotomy stems from its reliance solely on the midpoint of the search range to narrow this range, without fully leveraging the function value information at the interval endpoints to expedite the narrowing of the search range. In contrast, the false position algorithm utilizes linear interpolation of the function values at the search range endpoints (as depicted in Eq. (15)) to determine the temporary radius, resulting in a more efficient narrowing of the search range. When the center coordinates of the circle are fixed, the variation in volume fraction enclosed by the circular interface intersecting with the mixed grid cell across different radii is illustrated in Fig. 16. This trend of the volume fraction with respect to radius change assumes a curved shape, whereas the false position method excels in solving approximately linear functions. Consequently, relying solely on the false position method may inadvertently lead to an increase in the number of corrections required within the curved region. To address this issue, a hybrid dichotomy-false position method is utilized. Initially, the dichotomy is applied multiple times to narrow down the search range of the radius. When the trend of the volume fraction with respect to radius change approximates a linear shape within the search range, the calculation seamlessly transitions to the false position method, effectively reducing the number of corrections needed. Furthermore, the secant method is another efficient solution, leveraging the first two solutions and their corresponding function values to determine the subsequent solution. The specific computation procedure of the secant method is outlined in Eq. (16).

(14) $$\text{Dichotomy}\;\text{method}:R_{\text{temp}}=\frac{R_{\min}+R_{\max}}{2}$$

(15) $$\text{False}\;\text{position}\;\text{method}:R_{\text{temp}}=R_{\max}-\frac{c_{\max}-c^{*}}{c_{\min}-c_{\max}}\left(R_{\min}-R_{\max}\right)$$

(16) $$\text{Secant}\;\text{method}:R_{\text{temp}}=R_{i}-\left(c_{i}-c^{*}\right)\frac{R_{i}-R_{i-1}}{c_{i}-c_{i-1}}$$

where *R*_temp_ represents the temporary radius during the correction process; *R*_min_ and *R*_max_ respectively denote the left and right endpoint of the solving interval; *c*_min_ and *c*_max_ correspond to the volume fractions at these two endpoints; c* represents the exact volume fraction of the mixed grid cell; *c_i_* represents the calculated volume fraction at the current iteration using the secant algorithm, while *c_i_*_-1_ denotes the volume fraction of the previous iteration; *R_i_* and *R_i_*_-1_ represent the radius values at the current and prior iterations, respectively, during the correction process employing the secant method.

The hybrid dichotomy-false position and secant methods are used as correction methods in the strict mass conservation strategy. The distribution of corrections required for each method is also shown in Fig. 15. Both methods effectively reduce the number of corrections needed, with the hybrid method averaging 11 corrections and the secant method averaging 8 corrections. Given that the secant method requires the least corrections, it is chosen to correct the radius values predicted by the neural network models in the proposed strict mass conservation strategy.

## 3 Results and discussions

This section will comprehensively test the performance of the proposed CIR-ANN and CIR-CNN algorithms in reconstructing interfaces and capturing bubbles. The accuracies of both algorithms will be compared with those of other algorithms in these tests. Furthermore, the time cost of the CIR-ANN and CIR-CNN algorithms will also be discussed.

### 3.1 Brief introduction to other interface reconstruction algorithms

The PLIC algorithm approximates the vapor-liquid interface within each mixed grid cell using line segments, approximating the entire vapor-liquid interface as a piecewise linear interface. This algorithm requires determining both the normal vector and the constant term of the interface equation to specify the precise position of the linear interface. The normal vector can be computed in various ways, resulting in different PLIC-type linear interface reconstruction methods. Meanwhile, the constant term of the interface equation is determined based on the mass conservation constraint. The PLIC algorithm is a relatively simple and fast interface reconstruction algorithm.

The ELVIRA algorithm employs three different difference schemes of forward, backward, and central differences to calculate the slope of the interface based on the volume fractions in the *x* and *y*-axis directions within the 3 × 3 grid cell block. This process generates six candidate slopes. Subsequently, an error function related to the volume fractions is constructed, which indicates the deviation between the volume fractions of all grid cells in the 3 × 3 grid cell block calculated from the interface reconstructed by one candidate slope and the exact volume fractions. Among these six slopes, the one that yields the lowest error is chosen as the final slope for reconstructing the interface. Compared to the PLIC method, ELVIRA requires calculating six candidate slopes and determining the minimum value of the error function.

The QUASI algorithm first utilizes the PLIC algorithm to reconstruct the initial interface and then optimizes the reconstructed linear interface. The optimization involves adjusting the endpoint positions of the linear interface to ensure first-order continuity across all interfaces. Subsequently, the quadratic function is used to fit the endpoints of the linear interfaces within each mixed grid cell, resulting in a continuous curved interface. Following this, the second optimization is performed on the interface by adjusting the positions of the midpoints represented by the quadratic function, making the interface smooth and ensuring second-order continuity. Due to the two interface optimization processes and the quadratic function fitting in the QUASI algorithm, its reconstruction accuracy is relatively high. However, the implementation of the QUASI algorithm is relatively complex and time-consuming.

The VOSET-PLIC algorithm was mentioned to compare the accuracy of curve reconstruction when Chen et al. [28] proposed the CIR algorithm. The VOSET-PLIC and CIR algorithms use the VOSET algorithm to aid interface reconstruction. However, the CIR algorithm is based on the VOSET algorithm for curve interface reconstruction, while the VOSET-PLIC algorithm is based on the VOSET algorithm for linear interface reconstruction.

### 3.2 Static interface reconstruction

**Fig. 17:**
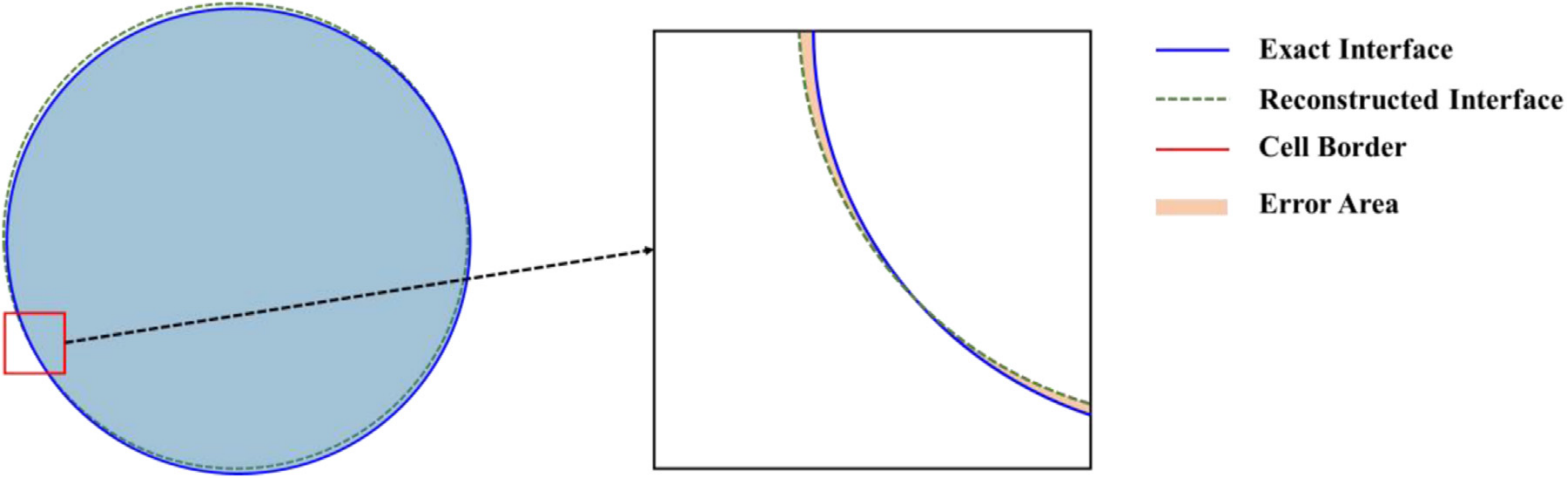
**Error area between the reconstructed interface and the exact interface**.

In this section, the CIR-ANN and CIR-CNN algorithms are employed to reconstruct 1000 random circular interfaces. Within a 1.0 × 1.0 computational domain, the centers of 1000 circular interfaces are near the center of the computational domain, and their radii are in the range of 0.2 to 0.25. The accuracy of the reconstructed interface is evaluated by calculating the *L*_1_ error using Eqs. (17) and (18).

(17) $$L_{1}=\frac{1}{l}\int \int |f\left(x,y\right)-\tilde{f}\left(x,y\right)|dxdy,$$

(18) $$f\left(x,y\right)=\left\{ \begin{array}{c} 1\;\text{if}\;\text{there}\;\text{is}\;\text{the}\;\text{main}\;\text{phase}\;\text{at}\;\text{the}\;\text{point}\left(x,y\right)\\ 0\;\text{if}\;\text{there}\;{\text{isn}}^{\prime }t\;\text{the}\;\text{main}\;\text{phase}\;\text{at}\;\text{the}\;\text{point}\left(x,y\right) \end{array}\right.$$

where *l* denotes the circumference of the circular interface, and the integral in Eq. (17) represents the error area between the reconstructed interface and the exact interface, as illustrated in Fig. 17.

**Fig. 18:**
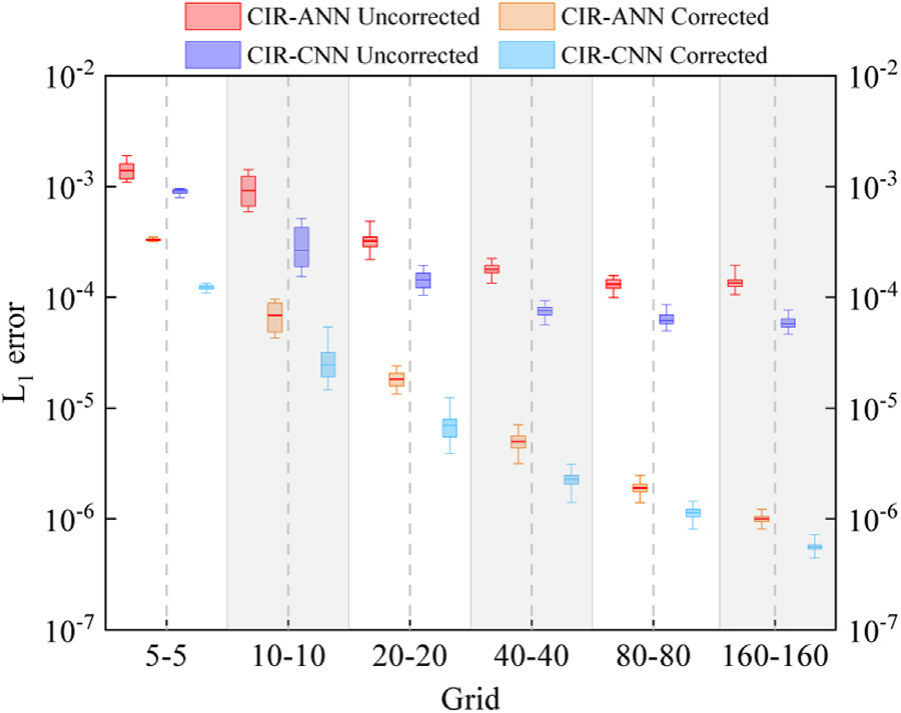
**Boxplot of L_1_ errors for neural network-based algorithms across different grid resolutions**.

**Fig. 19:**
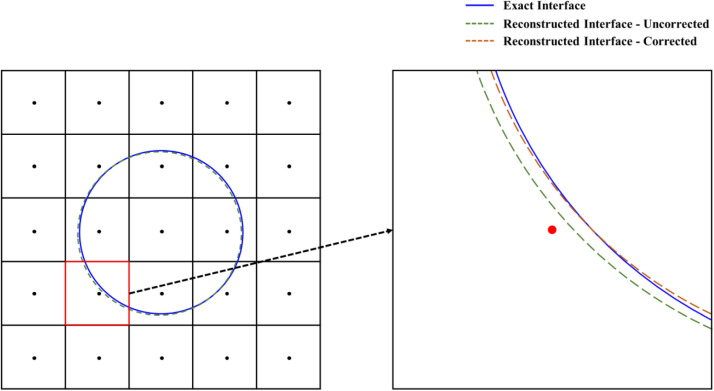
**Explanation of the strict mass conservation strategy for improving the accuracies of the proposed algorithms**.

The *L*_1_ errors of reconstructing 1000 random circular interfaces using the proposed neural network-based algorithms and other traditional algorithms are illustrated in Table 4. The uncorrected results indicate the predicted radius of the reconstructed curve interface is not correct using the strict mass conversation strategy. On the contrary, the correction process is required to derive the corrected results. Firstly, the difference in reconstruction accuracy between the CIR-ANN and CIR-CNN algorithms is illustrated. The CIR-CNN algorithm outperforms the CIR-ANN in reconstruction accuracy, which aligns with the better performance of the CNN model on the test dataset. Before the implementation of strict mass conservation strategies, the average accuracy of the CIR-CNN algorithm is 2.25 times that of the CIR-ANN algorithm across various grid resolutions. Implementing the strict mass conservation strategy significantly improves the accuracy of both algorithms. The change in algorithm accuracy before and after implementing the strategy is illustrated in Fig. 18. On a 5 × 5 grid, the improvement ratios in reconstruction accuracy for the CIR-ANN and CIR-CNN algorithms are 3.19 and 6.35, respectively. As the grid resolution increases, the improvement in accuracy resulting from the correction process in the strict mass conservation strategy becomes more pronounced. From a 5 × 5 grid to a 160 × 160 grid, the accuracy improvement ratios increase from 3.19 to 134.00 for the CIR-ANN algorithm and from 6.35 to 103.80 for the CIR-CNN algorithm. The reason for the better performance of the CIR-ANN and CIR-CNN algorithms after implementing the strict mass conservation strategies is illustrated as follows. As shown in Fig. 19, within a 5 × 5 grid, a circular interface with a radius of 0.2566274, centered at (0.5,0.5), intersects a grid cell centered at (0.3,0.3). The details of the exact and reconstructed interfaces are outlined in Table 5. Before implementing the strict mass conservation strategy, the circle center and radius predicted by the neural network exhibit a slight deviation from the exact ones. These errors collectively cause the reconstructed interface to deviate towards the lower left direction compared to the exact interface (as seen in Fig. 19(a)), ultimately resulting in a large area error between the two interfaces. After implementing the mass conservation strategy, the accuracy of the radius of the reconstructed curve interface is enhanced, leading to a significant decrease in the *L*_1_ error for the corrected interface. In the subsequent sections, unless stated otherwise, both neural network-based algorithms are assumed to have already implemented the strict mass conservation strategy.

**Table 4:** ***L*_1_ errors of different algorithms on the static interface reconstruction test**.

Grid	5 × 5	10 × 10	20 × 20	40 × 40	80 × 80	160 × 160
PLIC	–	1.37 × 10^–3^	3.60 × 10^–4^	1.20 × 10^–4^	5.04 × 10^–5^	2.32 × 10^–5^
ELVIRA	–	2.63 × 10^–3^	8.34 × 10^–4^	2.59 × 10^–4^	7.39 × 10^–5^	1.97 × 10^–5^
QUASI (after the first correction)	–	7.96 × 10^–4^	2.66 × 10^–4^	5.79 × 10^–5^	2.10 × 10^–5^	1.09 × 10^–5^
QUASI (after the second correction)	–	3.82 × 10^–5^	2.13 × 10^–6^	1.32 × 10^–7^	8.31 × 10^–9^	5.34 × 10^–10^
CIR	1.09 × 10^–3^	1.56 × 10^–4^	2.42 × 10^–5^	5.64 × 10^–6^	2.17 × 10^–6^	9.83 × 10^–7^
CIR-ANN (uncorrected)	1.39 × 10^–3^	9.19 × 10^–4^	3.23 × 10^–4^	1.80 × 10^–4^	1.32 × 10^–4^	1.35 × 10^–4^
CIR-ANN (corrected)	3.32 × 10^–4^	6.88 × 10^–5^	1.82 × 10^–5^	5.00 × 10^–6^	1.91 × 10^–6^	1.00 × 10^–6^
CIR-CNN (uncorrected)	9.04 × 10^–4^	3.05 × 10^–4^	1.45 × 10^–4^	7.59 × 10^–5^	6.35 × 10^–5^	5.89 × 10^–5^
CIR-CNN (corrected)	1.23 × 10^–4^	2.63 × 10^–5^	6.98 × 10^–6^	2.27 × 10^–6^	1.14 × 10^–6^	5.62 × 10^–7^

**Table 5:** **Parameters of the exact interface and reconstructed interface**.

	Exact interface	Reconstructed interface (uncorrected)	Reconstructed interface (corrected)
*a*	0.5000000	0.4996110	0.4996110
*b*	0.5000000	0.4998272	0.4998272
*r*	0.2566274	0.2575657	0.2562364

**Fig. 20:**
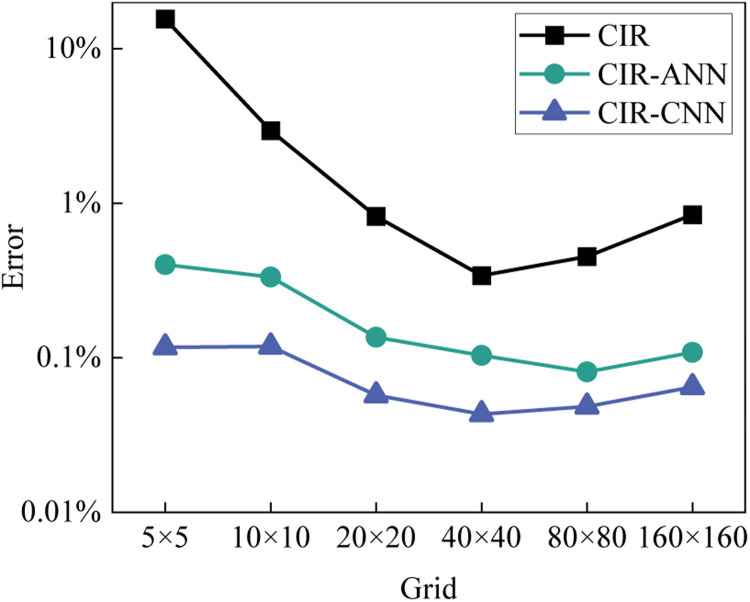
**Average errors in calculating the center coordinates and radii of the interfaces using different algorithms at different grid resolutions**.

Next, the comparison in reconstruction accuracy between the proposed algorithms and other traditional algorithms is demonstrated. Under different grid resolutions, the *L*_1_ errors of the QUASI, CIR, CIR-ANN, and CIR-CNN algorithms are significantly smaller than those of the PLIC and ELVIRA algorithms. This result demonstrates that the curve reconstruction methods have a distinct advantage in accuracy over traditional linear reconstruction methods. In a comprehensive comparison with the PLIC algorithm, ELVIRA algorithm, QUASI (after the first correction), and CIR algorithm, the proposed CIR-ANN algorithm shows average accuracy ratios of 22.66, 38.85, 11.93, and 1.69, respectively. The CIR-CNN algorithm achieves even higher accuracy, with average ratios of 48.40, 86.69, 26.34, and 4.07, respectively. The CIR, CIR-ANN, and CIR-CNN algorithms all use a portion of a standard circle to approximate the vapor-liquid interface within the mixed grid cell. While the CIR algorithm uses the numerical algorithm to determine the parameters of the standard circle equation, the CIR-ANN and CIR-CNN algorithms use well-trained neural network models to provide the parameters. Compared to the CIR algorithm, the CIR-ANN and CIR-CNN algorithms notably show a higher accuracy, with average accuracy ratios of 1.69 and 4.07, respectively. The accuracy improvements are more pronounced when tested on a coarse grid. Specifically, on a 5 × 5 grid, the accuracy ratios for the CIR-ANN and CIR-CNN algorithms are 3.28 and 8.86, respectively. As the grid resolution increases, the improvement accuracy of the CIR-ANN and CIR-CNN algorithms compared to the CIR algorithm gradually decreases. On the 160 × 160 grid, the accuracy ratio of CIR-ANN is 0.98, while the accuracy ratio of CIR-CNN is 1.75. The superior accuracy of the CIR-ANN and CIR-CNN algorithms, when compared to the CIR algorithm, is primarily attributed to their ability to predict the circle center coordinates and radii more precisely, particularly on coarse grids, as demonstrated in Fig. 20. These results indicate that both the CIR-ANN and CIR-CNN algorithms proposed in this paper have a significant advantage in improving reconstruction accuracy. It should be noted that the QUASI algorithm, after the second interface optimization, has the highest accuracy among these algorithms, primarily due to its second interface optimization process. However, the high accuracy of QUASI comes at the cost of longer processing time, which will be explained in the subsequent comparison of time costs with other algorithms.

**Fig. 21:**
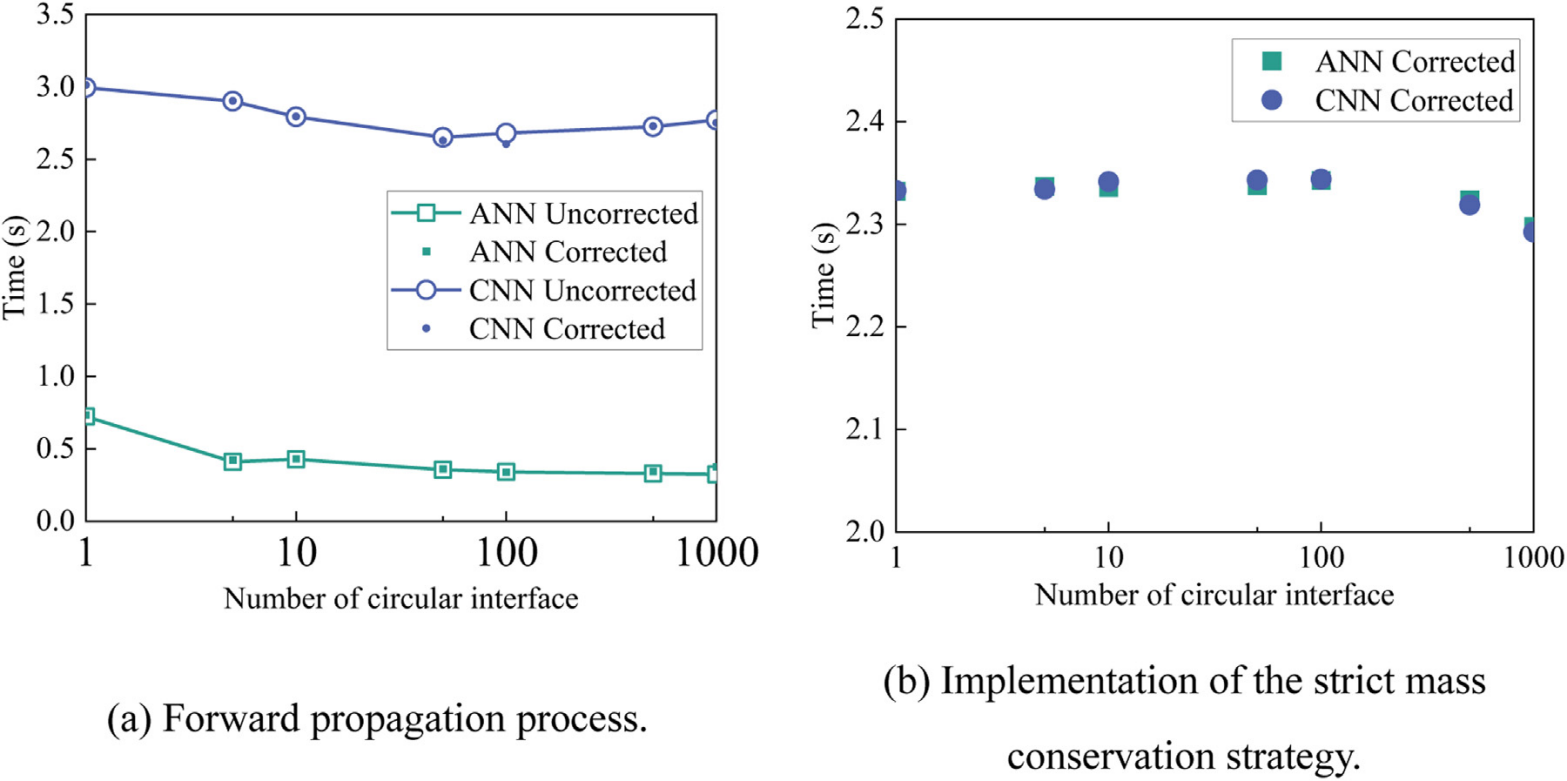
**Time costs of (a) the forward propagation of neural network models and (b) implementing the strict mass conservation strategy**.

The time costs of the CIR-ANN and CIR-CNN algorithms primarily consist of two parts: the time required for the forward propagation process of the neural network model and the time spent on implementing the strict mass conservation strategy. A significant advantage of neural networks is their ability to process large amounts of data in parallel, accelerating the prediction process. In this paper, the neural networks are employed to simultaneously perform the prediction process of the parameters of all mixed grid cells intersected with a circular interface. To fully exploit this advantage of neural networks, the neural network models in the CIR-ANN algorithm and CIR-CNN algorithm are used to predict parameters of a varying number of circular interfaces simultaneously, specifically 1, 5, 10, 50, 100, 500, and 1000 interfaces. It is crucial to clarify that the total number of circular interfaces remains 1000. For instance, if the neural network simultaneously predicts the parameters of the 100 circular interfaces, and the total number of interfaces is 1000, then the prediction process would need to be repeated 10 times to cover all interfaces. The time costs of using neural network models to predict parameters of various numbers of circular interfaces simultaneously on a 160 × 160 grid are shown in Fig. 21. It is important to note that the time costs of the CIR-ANN and CIR-CNN algorithms are measured on a desktop computer with an Intel(R) Xeon(R) W-2125 CPU operating at 4.00 GHz and an NVIDIA GeForce GTX 1080 Ti graphics card.

As the number of interfaces simultaneously predicted by neural network models increases, the time cost for the forward computation process shows a trend of initially decreasing and then increasing. This is because when neural network models process small batch data inputs, they cannot fully utilize computational resources and the parallel computing capabilities of neural networks, resulting in longer processing times. With an increase in batch data inputs, computational resources are better utilized, and the parallel computing capabilities of neural networks are fully used, leading to an overall decrease in processing time. However, when the batch size exceeds a certain threshold, computational resources and the parallel computing capabilities of neural networks reach saturation. As a result, factors such as computer memory bandwidth and cache capacity begin to limit the computing capabilities of neural network models, leading to a further increase in processing time.

Additionally, it is worth noting that as the number of batches increases, the time variation in implementing the strict mass conservation strategy is not significant. This is because regardless of how the batch number changes, the total number of interfaces to be calculated remains constant. Although the radii of these 1000 circular interfaces are randomly varied in the range of 0.2 to 0.25, the total number of mixed grid cells to be processed for the 1000 circular interfaces is not very variable. It is approximately equal to the number of mixed grid cells to be processed for 1000 circular interfaces with a radius of 0.225. Furthermore, well-trained ANN and CNN models require approximately the same number of corrections for a mixed grid cell. As a result, the time costs for CIR-ANN and CIR-CNN algorithms to implement the strict mass conservation strategy are very similar. These time costs do not change significantly with increasing batch data size.

It may also be noted that the forward propagation process of the CNN model of the CIR-CNN algorithm takes longer, averaging approximately 7 times the time consumption of the ANN model of the CIR-ANN algorithm. This increased time consumption stems from the intricate structure of CNN, leading to higher computational complexity during processing data of the same size. The floating-point operations (FLOPs) can be calculated based on the specific architectures of the ANN and CNN adopted in the CIR-ANN and CIR-CNN algorithms shown in Appendix A, which reflect the computational complexity of the neural network models. The FLOPs of the CNN model are approximately 5.86 times that of the ANN model, with 809,760 FLOPs for the CNN model and 138,240 FLOPs for the ANN model, respectively. Although the forward propagation process of the CNN model in the CIR-CNN algorithm requires more time for processing, the CIR-CNN algorithm achieves greater accuracy. When varying numbers of circular interfaces are simultaneously input to the neural network models in each batch, implementing the strict mass conservation strategy accounts for an average of 84.59% of the total time cost of the CIR-ANN algorithm. In contrast, within the CIR-CNN algorithm, implementing the same strategy accounts for an average of 45.67% of the total time cost. In both algorithms, the time required to implement the strict mass conservation strategy accounts for a large proportion. Implementing the strict mass conservation strategy cannot be carried out in parallel as in neural networks, and the implementation time is relatively long. Therefore, implementing the strict mass conservation strategy increases the time cost and consequently decreases the efficiency of the algorithms.

When the number of circular interfaces processed simultaneously by the neural network in each batch is set to 50, both ANN and CNN models require relatively less time for their forward propagation processes. Therefore, a batch size 50 is chosen for CIR-ANN and CIR-CNN algorithms when reconstructing 1000 random circular interfaces. Additionally, the time costs for reconstructing 1000 random circular interfaces using neural network-based algorithms and other algorithms are listed in Table 6. The numbers in parentheses indicate the time cost by both algorithms when implementing the strict mass conservation strategy. Although implementing the strict mass conservation strategy is time-consuming and accounts for a large portion of the total time cost, the overall time cost of the proposed algorithms remains lower compared to other algorithms (except the PLIC). As shown in Table 6, compared to the ELVIRA, QUASI (after the second correction), and CIR algorithms, the proposed algorithms require less time. Specifically, the average time reduction ratios are 10.20, 44.33, and 5.41 when using the CIR-ANN algorithm and 5.51, 23.95, and 2.91 when utilizing the CIR-CNN algorithm. While the PLIC algorithm may excel in simplicity, leading to the shortest implementation time, the proposed CIR-ANN and CIR-CNN algorithms achieve superior accuracy while maintaining a relatively short computation time.

**Table 6:** **Time costs for reconstructing 1000 circular interfaces**.

Grid	40 × 40	80 × 80	160 × 160
PLIC (Youngs's method)	0.44 s	0.92 s	3.26 s
ELVIRA	6.74 s	13.54 s	28.19 s
QUASI			
(after the second correction)	25.25 s	52.79 s	151.01 s
CIR	5.15 s	6.30 s	10.28 s
CIR-ANN (Uncorrected)	0.09 s	0.18 s	0.36 s
CIR-ANN (Corrected)	0.66 (0.57) s	1.36 (1.18) s	2.70 (2.33) s
CIR-CNN (Uncorrected)	0.69 s	1.35 s	2.65 s
CIR-CNN (Corrected)	1.24 (0.56) s	2.50 (1.19) s	4.97 (2.34) s

In conclusion, compared to other existing interface reconstruction algorithms, the CIR-ANN and CIR-CNN algorithms have demonstrated good performance in terms of computational accuracy. Despite the additional time required to implement the strict mass conservation strategy, the CIR-ANN and CIR-CNN algorithms maintain high efficiency. Consequently, the CIR-ANN and CIR-CNN algorithms are highly appealing curved reconstruction algorithms.

### 3.3 Dynamic interface reconstruction

In this section, the proposed CIR-ANN and CIR-CNN algorithms are tested on reconstructing dynamic interfaces under various flow fields, including horizontal translation, rotation, and complex time-reversed vortex. The CIR algorithm mentioned earlier is an efficient and accurate curved reconstruction algorithm. The VOSET-PLIC algorithm, a linear reconstruction algorithm, serves as a benchmark for Chen et al. [28] to compare the accuracy of the CIR algorithm. In the subsequent dynamic interface reconstruction testing process, the CIR-ANN and CIR-CNN algorithms will mainly be compared with the CIR and VOSET-PLIC algorithms regarding their performance. The performance of different reconstruction algorithms is evaluated in these tests using the deformation error, as defined in Eq. (19).

(19) $$D_{err}=\frac{\sum_{i,j}\left|c_{i,j}-c_{i,j}^{*}\right|}{\sum_{i,j}c_{i,j}^{*}}$$

where $c_{i,j}$ represents the calculated volume fraction of grid cell (*i, j*) obtained using the algorithms proposed in this paper; $c_{i,j}^{*}$ represents the exact volume fraction of the same grid cell (*i, j*).

During the test processes, the CIR-ANN and CIR-CNN algorithms are utilized for interface reconstruction, and the calculation of the VOF flux employs the same approach as detailed in the paper [28].

#### 3.3.1 Horizontal translation test

**Fig. 22:**
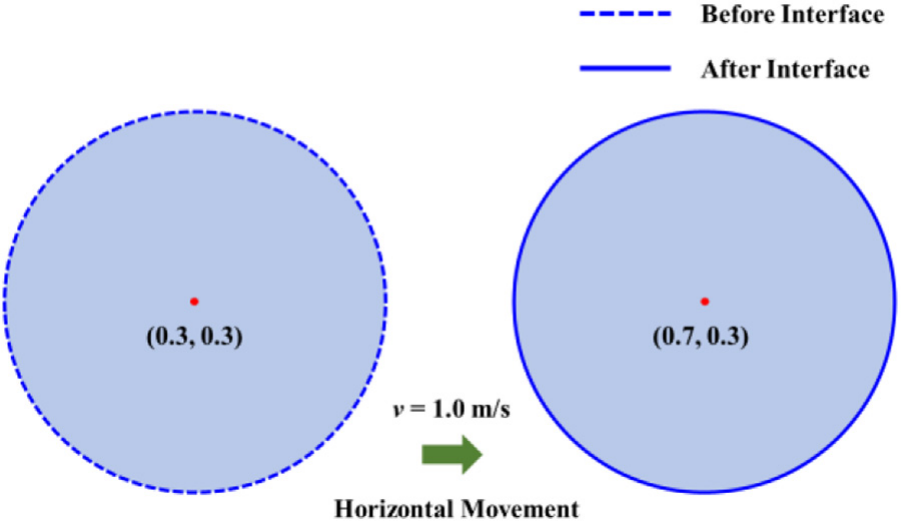
**Horizontal translation of a bubble**.

As shown in Fig. 22, a circular bubble with a radius of 0.2 is initially positioned at (0.3, 0.3) within a 1.0 × 1.0 computational domain and then horizontally moves to (0.7, 0.3) at a velocity of 1.0 m/s. The deformation errors of the final circular interface obtained by different interface reconstruction algorithms are presented in Table 7. Compared to VOSET-PLIC and CIR algorithms, CIR-ANN and CIR-CNN exhibit lower deformation errors. Compared to the VOSET-PLIC and CIR algorithms, the CIR-ANN algorithm shows average accuracy ratios of 29.01 and 4.14, while the CIR-CNN algorithm achieves average ratios of 102.36 and 12.11. It is also noticeable that CIR-ANN and CIR-CNN algorithms have higher accuracy ratios on coarser grids than VOSET-PLIC and CIR algorithms. As the grid becomes denser, the accuracy ratios of CIR-ANN and CIR-CNN gradually decrease. This is because the relative radius of circular interfaces is smaller at lower-resolution grids. The differences in volume fraction distributions formed by circular interfaces of different radii within grid cell blocks are more pronounced, allowing neural networks to identify circular interfaces of different radii better, thus providing higher accuracy in predicting the center coordinates and radii. As the grid resolution increases, the relative radii of circular interfaces become larger. The portion of the circular interface inside mixed grid cells gradually approaches a linear interface, making the interface shape with different radii closer. This results in less distinct differences in volume fraction distributions within grid cell blocks, causing the circle center coordinates and radii predicted by neural networks to be less accurate than those of the lower-resolution grids.

**Table 7:** **Deformation errors of different algorithms in the horizontal movement test**.

Grid	VOSET-PLIC	CIR	CIR-ANN	CIR-CNN
12 × 12	3.70 × 10^–2^	2.71 × 10^–3^	6.27 × 10^–4^	1.65 × 10^–4^
18 × 18	1.93 × 10^–2^	7.04 × 10^–4^	3.91 × 10^–4^	9.83 × 10^–5^
25 × 25	9.92 × 10^–3^	1.08 × 10^–3^	2.06 × 10^–4^	4.17 × 10^–5^
50 × 50	2.74 × 10^–3^	8.66 × 10^–4^	8.59 × 10^–5^	3.58 × 10^–5^
100 × 100	1.09 × 10^–3^	3.39 × 10^–4^	9.26 × 10^–5^	2.95 × 10^–5^
150 × 150	6.38 × 10^–4^	1.62 × 10^–4^	3.41 × 10^–5^	3.07 × 10^–5^
200 × 200	3.88 × 10^–4^	9.18 × 10^–5^	4.69 × 10^–5^	2.25 × 10^–5^
250 × 250	2.63 × 10^–4^	6.95 × 10^–5^	5.36 × 10^–5^	2.95 × 10^–5^

Under the grid resolutions of 100 × 100 and 200 × 200, the time costs of different algorithms are listed in Table 8. The CIR-ANN and CIR-CNN algorithms demonstrate significantly lower time costs than the VOSET-PLIC and the CIR algorithms. Specifically, the CIR-ANN algorithm consumes an average of 14.47% of the total time cost of the VOSET-PLIC algorithm, while the CIR-CNN algorithm consumes an average of 17.93%. Meanwhile, the CIR-ANN algorithm takes an average of 23.34% of the total time of the CIR algorithm, and the CIR-CNN algorithm takes an average of 30.24%. The efficient of the CIR-ANN and CIR-CNN is mainly due to the efficient computing capabilities of neural network models. The CIR-ANN algorithm takes slightly shorter than the CIR-CNN algorithm due to the relatively simple structure of the ANN model compared to that of the CNN model.

**Table 8:** **Time costs of different algorithms on the horizontal test**.

Grid	100 × 100	200 × 200
VOSET-PLIC	–	65.7 s
CIR	18.9 s	35.8 s
CIR-ANN	3.80 s	9.51 s
CIR-CNN	5.21 s	11.78 s

In conclusion, the CIR-ANN and CIR-CNN algorithms demonstrate higher computational accuracy and efficiency than the VOSET-PLIC and CIR algorithms in horizontal translation test. The CIR-ANN and CIR-CNN algorithms exhibit accuracy ratios of 29.01 and 102.36, respectively, compared to the VOSET-PLIC algorithm. In contrast, compared to the CIR algorithm, the average accuracy ratios are 4.14 and 12.11, respectively. In terms of efficiency, both the CIR-ANN and CIR-CNN algorithms require less than one-fifth of the time cost of the VOSET-PLIC algorithm, and both algorithms also require less than one-third of the time of the CIR algorithm.

#### 3.3.2 Zalesak rotation test

**Fig. 23:**
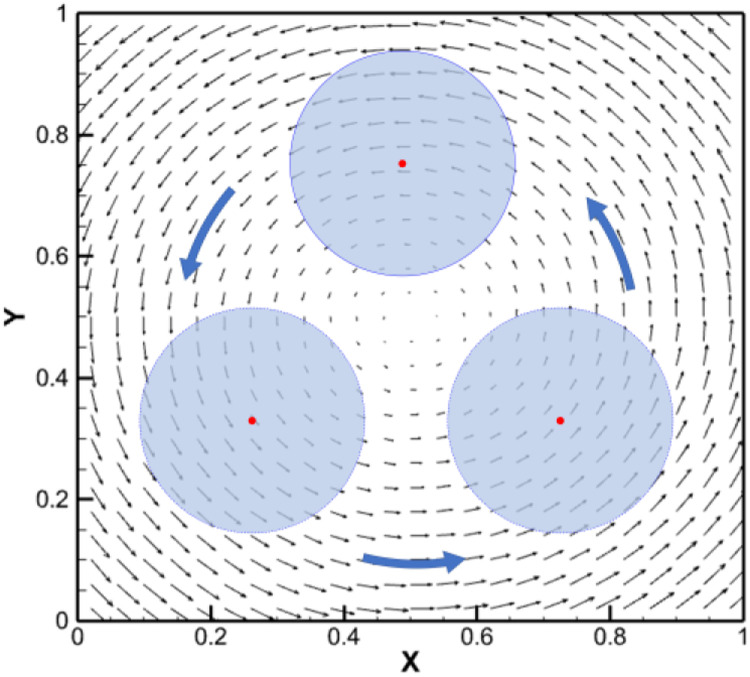
**Zalesak rotation of a bubble**.

As shown in Fig. 23, a circular bubble with its center at (0.5, 0.75) and a radius of 0.2 is located within the constant rotational velocity field described in Eqs. (20) and (21) will return to its original position after 4π seconds.

(20) $$u\left(x,y\right)=\frac{\left(0.5-y\right)}{2}$$

(21) $$v\left(x,y\right)=\frac{\left(x-0.5\right)}{2}$$

where u and *v* represent the velocity components in the *x*- and *y*-directions, respectively.

The deformation errors of the proposed algorithms, VOSET-PLIC and the CIR algorithm, are listed in Table 9. Compared to the VOSET-PLIC algorithm, the accuracy ratios of the CIR-ANN and CIR-CNN algorithms are an average of 2.40 and 2.40, respectively. However, the CIR-ANN and CIR-CNN algorithms are almost the same as the CIR algorithm across various grid resolutions. It is noteworthy that the accuracy of the proposed algorithms is not higher than that of the CIR algorithm as in the previous tests. It may arise from using the method to calculate VOF flux, leading to repeated calculations in certain areas of the VOF flux process. This repeated calculation could introduce errors in the computed VOF values. These errors gradually accumulate during the rotation process, thus leading to errors in the center coordinates and radius predicted by the neural network models based on these flawed volume fractions within the grid cell block. Nonetheless, the CIR-ANN and CIR-CNN algorithms still maintain a high level of accuracy, as does the CIR algorithm.

**Table 9:** **Deformation errors of different algorithms on the Zalesak rotation test**.

Grid	25 × 25	50 × 50	100 × 100	150 × 150	200 × 200
VOSET-PLIC	2.90 × 10^–2^	7.23 × 10^–3^	2.59 × 10^–3^	2.21 × 10^–3^	2.20 × 10^–3^
CIR	4.13 × 10^–3^	3.62 × 10^–3^	2.50 × 10^–3^	2.15 × 10^–3^	2.18 × 10^–3^
CIR-ANN	4.23 × 10^–3^	3.33 × 10^–3^	2.60 × 10^–3^	2.23 × 10^–3^	2.22 × 10^–3^
CIR-CNN	4.23 × 10^–3^	3.32 × 10^–3^	2.56 × 10^–3^	2.22 × 10^–3^	2.21 × 10^–3^

#### 3.3.3 Time-reversed vortex test

**Fig. 24:**
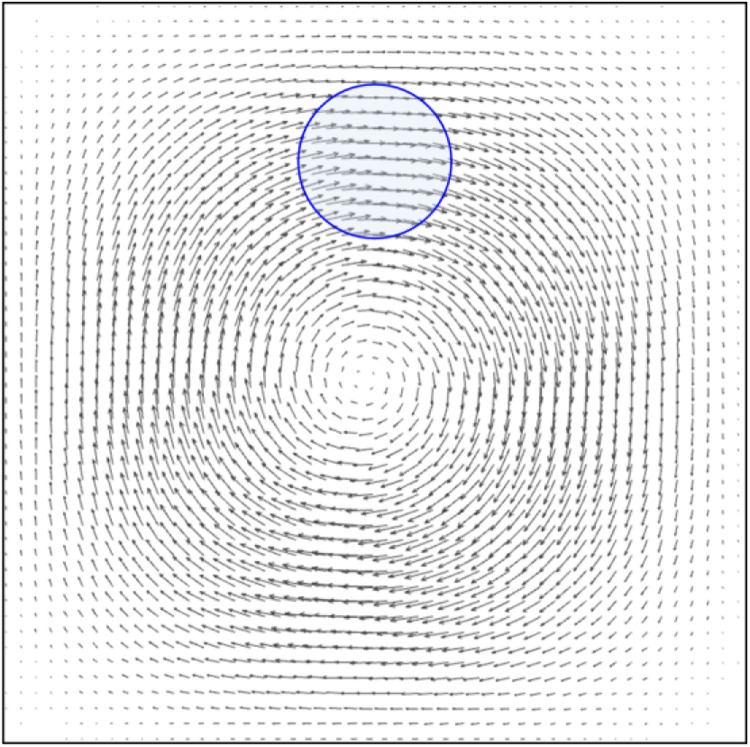
**The initial position of a bubble on the time-reversed vortex field**.

As shown in Fig. 24, a circular bubble with a radius of 0.15 is located at the initial position (0.5, 0.75). Within the computational domain, a vortex field continuously changes over time, with the velocity components in different directions defined by Eqs. (22) and (23). Under the shear of the time-reversed vortex field, the bubble will deform continuously and reach its maximum deformation after half a period. Subsequently, the vortex field reverses, gradually causing the bubble to return to its original position. Ultimately, starting from the moment of maximum deformation, the bubble returns to its original position after 4.0 s.

(22) $$u\left(x,y,\tau \right)=-\sin\left(2\pi y\right)\sin^{2}\left(\pi x\right)\cos\left(\pi \tau /T\right)$$

(23) $$v\left(x,y,\tau \right)=\sin\left(2\pi x\right)\sin^{2}\left(\pi y\right)\cos\left(\pi \tau /T\right)$$

where u and *v* are the velocity components in different directions; *x* and *y* are the coordinates of the center of the grid cell; *τ* denotes time; and *T* is the period of change of the vortex field, 8.0 s.

**Fig. 25:**
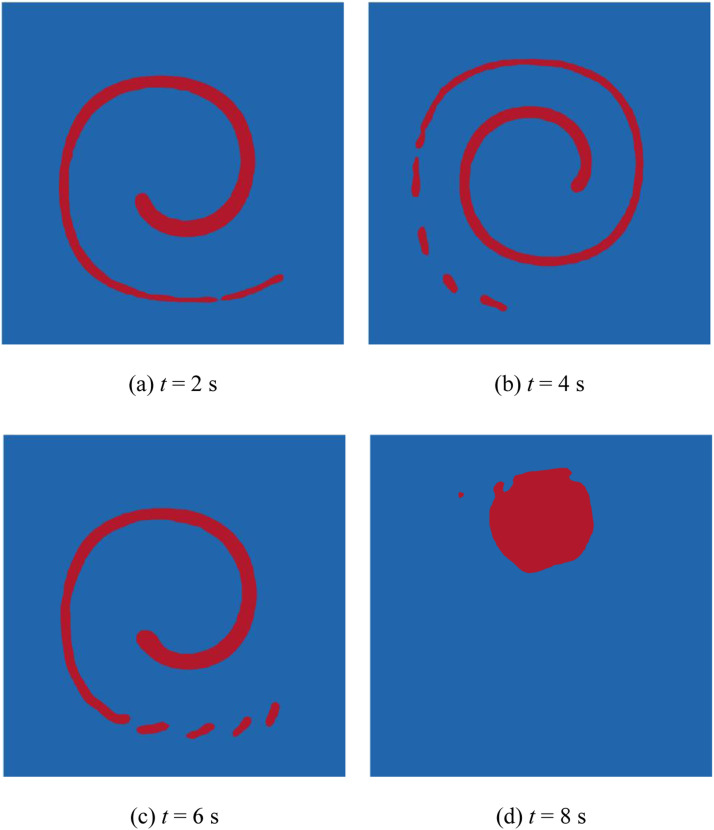
**Deformation of bubbles at (a) 2 s, (b) 4 s, (c) 6 s, and (d) 8 s using the proposed CIR-ANN algorithm at 100 × 100 grid resolution**.

**Fig. 26:**
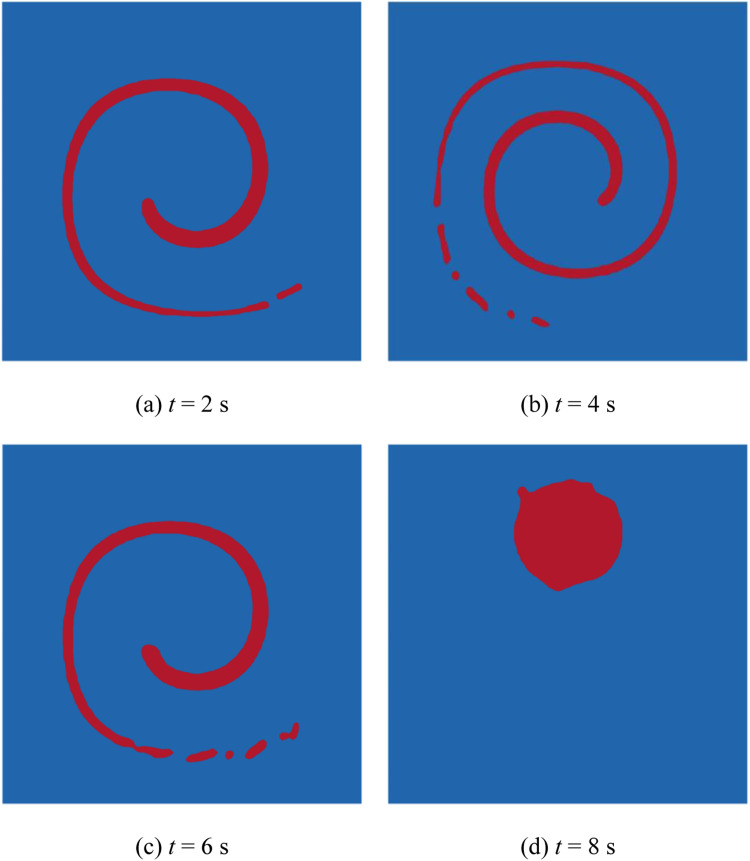
**Deformation of bubbles at (a) 2 s, (b) 4 s, (c) 6 s, and (d) 8 s using the proposed CIR-CNN algorithm at 100 × 100 grid resolution**.

The deformation of the bubbles at different times under the time-reversed vortex field is shown in Fig. 25, Fig. 26. The bubbles undergo complex deformation due to the vortex field. The distribution of volume fractions within the grid cell block, which serves as input to the neural network, becomes highly complex. There is a significant increase in the discrepancy between the data obtained from the vortex field and the train dataset, potentially leading to a notable decrease in the prediction accuracy of the neural networks.

**Fig. 27:**
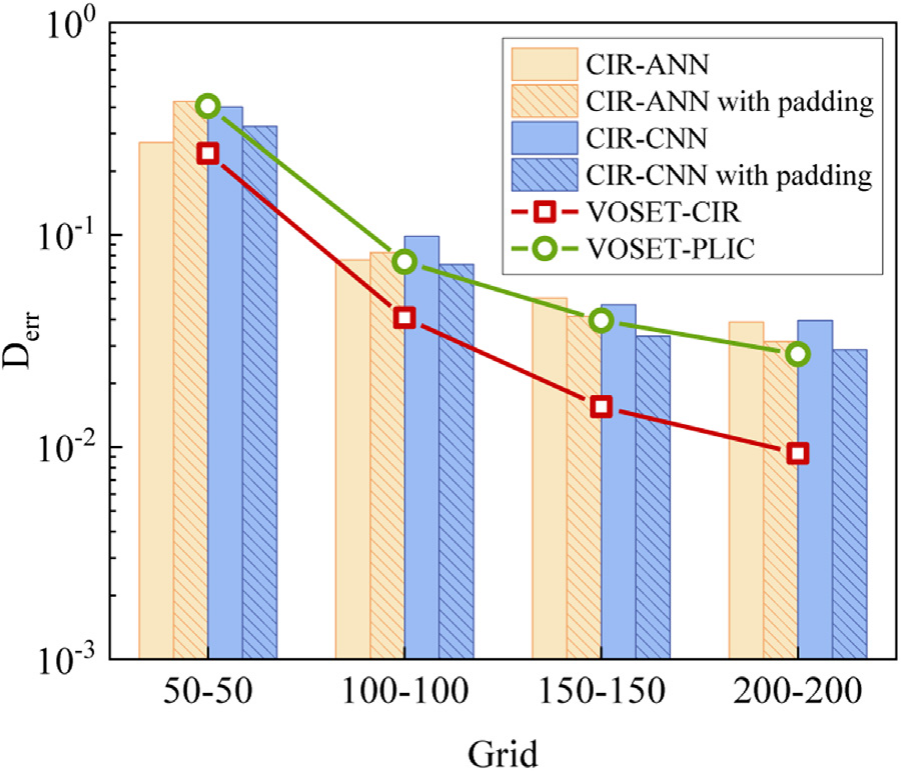
**Deformation errors of the bubble returning to its original position under different grid resolutions**.

Fig. 27 and Table 10 present the performance of CIR-ANN and CIR-CNN algorithms in capturing the bubble in a time-reversed vortex field. Under different resolution grids, both CIR-ANN and CIR-CNN algorithms exhibit lower accuracy than the CIR algorithm. This accuracy gap tends to widen slightly as the grid resolution increases. However, it is noteworthy that the accuracies of these two proposed algorithms are comparable to the VOSET-PLIC algorithm. Although the accuracies of the CIR-ANN and CIR-CNN algorithms decrease in the complex vortex field characterized by complex fluid volume fraction distributions, they remain within an acceptable accuracy level due to the advantages of curved reconstruction.

**Table 10:** **Deformation errors of different algorithms on the time-reversed vortex test**.

Grid	50 × 50	100 × 100	150 × 150	200 × 200
CIR	2.42 × 10^–1^	4.07 × 10^–2^	1.55 × 10^–2^	9.32 × 10^–3^
VOSET-PLIC	4.05 × 10^–1^	7.47 × 10^–2^	3.95 × 10^–2^	2.74 × 10^–2^
CIR-ANN	2.72 × 10^–1^	7.62 × 10^–2^	5.04 × 10^–2^	3.88 × 10^–2^
CIR-ANN with padding	4.26 × 10^–1^	8.26 × 10^–2^	4.14 × 10^–2^	3.15 × 10^–2^
CIR-CNN	4.01 × 10^–1^	9.83 × 10^–2^	4.70 × 10^–2^	3.95 × 10^–2^
CIR-CNN with padding	3.25 × 10^–1^	7.25 × 10^–2^	3.33 × 10^–2^	2.88 × 10^–2^

Notably, the CIR-ANN algorithm exhibits higher accuracy at lower grid resolutions than the CIR-CNN algorithm. It is inconsistent with the usual superior performance of the CIR-CNN algorithm in previous tests. Specifically, at grid resolutions of 50 × 50 and 100 × 100, compared to the CIR-CNN algorithm, the accuracy ratio of the CIR-ANN algorithm is an average of 1.38. As the grid resolution increases, the difference in accuracy between the CIR-ANN and CIR-CNN algorithms gradually decreases. However, at grid resolutions 150 × 150 and 200 × 200, the accuracy ratio of the CIR-ANN algorithm, compared to the CIR-CNN algorithm, is an average of 0.98, indicating that its accuracy is slightly lower than that of the CIR-CNN algorithm at these resolutions. Overall, across those grid resolutions, the CIR-ANN algorithm demonstrates an average accuracy ratio of 1.18 compared to the CIR-CNN algorithm. As mentioned earlier, the input data for the ANN model of the CIR-ANN algorithm consists of volume fractions that have been flattened. Consequently, the spatial relationship between the volume fractions input to the ANN model is weaker compared to the input data of the CNN model. In contrast, the volume fraction distribution in a complex flow field is highly complex, indicating a complex spatial relationship. This complex spatial relationship may challenge the neural network to process and result in significant errors. However, the ANN model used in the CIR-ANN algorithm is less affected by the complex spatial relations due to the flattening operation of the input data. Therefore, CIR-ANN shows higher accuracy than the CIR-CNN algorithm in this test.

Adopting a 5 × 5 grid cell block may contain multiple interfaces, especially in coarse grids, which may lead to unsatisfactory performance of proposed algorithms. To improve the accuracy of the proposed algorithms, a method of selectively filling the volume fractions of grid cells within the grid cell block has been proposed. Specifically, within the grid cell block, when a change in the monotonicity of the volume fraction of the grid cell block within the columns or rows is detected, the corresponding grid cell is filled. The detailed preprocessing filling procedure can be found in Appendix B. As shown in Fig. 27, the method of pre-filling grid cell volume fraction has improved the accuracy of the CIR-CNN algorithm. However, it is worth noting that the impact of the pre-filling method on the CIR-ANN algorithm is complex. Specifically, at grid sizes of 50 × 50 and 100 × 100, pre-filling has harmed the accuracy of the CIR-ANN algorithm. Conversely, the method has shown positive effects at grid resolutions of 150 × 150 and 200 × 200.

In this section, the accuracy of the CIR-ANN and CIR-CNN algorithms decreases due to the complexity of the time-reversed vortex field. The result of the time-reversed vortex field test reveals that the accuracies of the CIR-ANN and CIR-CNN algorithms are slightly lower than that of the CIR algorithm but comparable to the accuracy of the VOSET-PLIC algorithm. Overall, both algorithms demonstrate satisfactory performance.

## 4 Conclusion

Based on artificial neural network (ANN) and convolutional neural network (CNN), two curved interface reconstruction algorithms, namely CIR-ANN and CIR-CNN, are proposed. The vapor-liquid interface within a mixed grid cell is approximated by CIR-ANN and CIR-CNN algorithms using a portion of a circle. Notably, the neural network models integrated within the CIR-ANN and CIR-CNN algorithms can output the center coordinates and radius of this approximation circle with high precision. Compared to other interface reconstruction algorithms, the proposed CIR-ANN and CIR-CNN algorithms demonstrate significant accuracy and computational efficiency advantages. The main conclusions of this paper can be summarized as follows:

(1) Two interface reconstruction algorithms based on ANN and CNN, namely CIR-ANN and CIR-CNN, are proposed in this paper. These neural networks, employed in both algorithms, take the volume fractions within a 5 × 5 grid cell block as input to predict the center coordinates and radius of a circle, a portion of this circle being used to approximate the vapor-liquid interface within the central mixed grid cell of the block. To strictly satisfy the mass conservation constraint, a strategy is proposed that ensures strict mass conservation. This strategy employs an efficient secant method to adjust the radii the neural networks predicted moderately. With the implementation of the strict mass conservation strategy, the CIR-ANN and CIR-CNN algorithms can achieve high-accuracy interface reconstruction while balancing the advantage of neural networks.
(2) The CIR-ANN and CIR-CNN algorithms have high accuracy in static interface reconstruction. One thousand random circular interfaces are constructed using the CIR-ANN and CIR-CNN algorithms. Compared to the PLIC algorithm, ELVIRA algorithm, QUASI algorithm (after the first correction), and CIR algorithm, the CIR-ANN algorithm exhibits average accuracy ratios of 22.66, 38.85, 11.93, and 1.69, while the CIR-CNN algorithm shows accuracy ratios of 48.40, 86.69, 26.34, and 4.07 across different grid resolutions. Compared to the QUASI algorithm with twice interface optimizations, the accuracies of the CIR-ANN and CIR-CNN are lower. Still, owing to their simplicity and efficiency, the time costs of reconstructing random circular interfaces are shorter.
(3) The CIR-ANN and CIR-CNN algorithms also demonstrate favorable performance in dynamic interface reconstruction. In the horizontal interface movement test, compared to the CIR algorithm, the proposed algorithms achieve significant improvements in accuracy, with average ratios of 4.14 for the CIR-ANN algorithm and 12.11 for the CIR-CNN algorithm. In the rotation test, the computational accuracies of the proposed algorithms are comparable to those of the CIR algorithm. Furthermore, in the time-reversed vortex field test, due to the complexity of the flow fields, an input data filling strategy is proposed to reduce the discrepancy between actual data and the train dataset, thereby mitigating the influence of the potential prediction failures of the neural network models. Although the proposed algorithms exhibit slightly lower average accuracy than the CIR algorithm in the time-reversed vortex field test, each of their accuracies is comparable to the accuracy of the VOSET-PLIC algorithm.
(4) The CIR-ANN and CIR-CNN also favor reducing the computational time costs when used for interface reconstruction. In the reconstruction of random circular interfaces, compared to the ELVIRA algorithm, QUASI (after the second correction) algorithm, and CIR algorithm, the ANN-based algorithm reduces average processing time with reduction ratios of 10.20, 44.33, and 5.41, while the CNN-based algorithm achieves reductions of 5.51, 23.95, and 2.91. In a horizontal movement test, both the CIR-ANN and CIR-CNN algorithms require less than one-fifth of the time cost of the VOSET-PLIC algorithm, and both algorithms also require less than one-third of the time of the CIR algorithm.

## Declaration of competing interests

The authors declare that they have no conflicts of interest in this work.

## Figures and Tables

**Table 11:** Finalized structure of the ANN model.

Component	Operation	Output data shape
Input	–	N × 25
Linear Layer 1	Linear/GELU	N × 128
Linear Layer 2	Linear/GELU	N × 256
Linear Layer 3	Linear/GELU	N × 128
Linear Layer 4	Linear/GELU	N × 3
Output	–	N × 3

**Table 12:** Finalized structure of the CNN model.

Component	Operation	Output data shape
Input	–	N × 1 × 5 × 5
Convolutional layer 1	Conv2d/GELU	N × 35 × 4 × 4
Convolutional layer 2	Conv2d/GELU	N × 70 × 3 × 3
Convolutional layer 3	Conv2d/GELU	N × 140 × 2 × 2
Convolutional layer 4	Conv2d/GELU	N × 280 × 1 × 1
Reshape	–	N × 280
Linear layer 1	Linear	N × 3
Output	–	N × 3

**Fig. 28:**
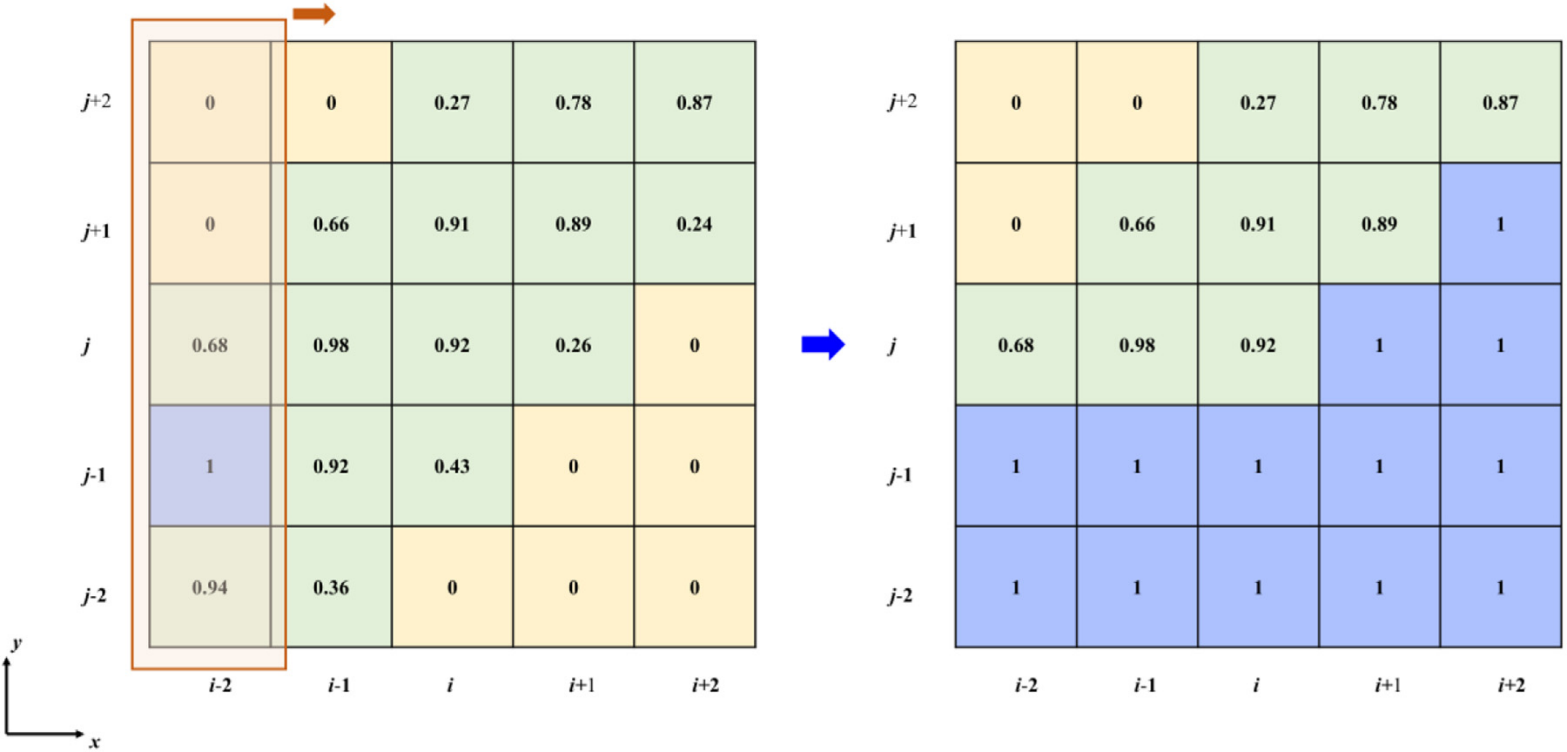
**Downward filling scenario**.

**Fig. 29:**
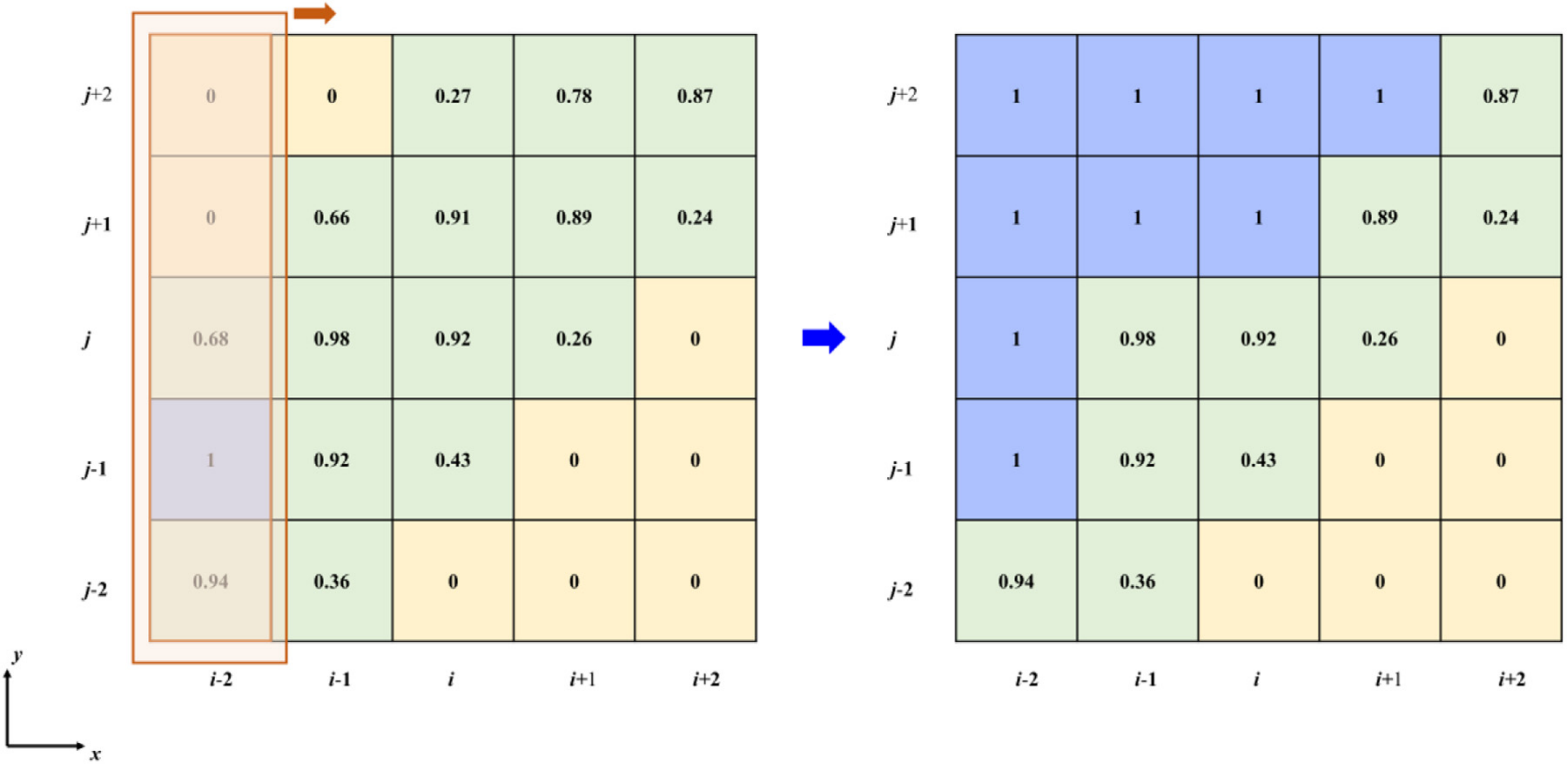
**Upward filling scenario**.

## Appendix A Architecture of the ANN and CNN models

The ANN and CNN structures utilized in this paper are presented in Table 11 and Table 12, respectively. "Linear" refers to the linear transformation operation performed within a linear layer in ANN and CNN models. "Conv2d" refers to the 2D convolutional operation in convolutional layers, which convolves the input with a kernel of a specified size, with parameters like stride and padding determining the output dimensions. Unless otherwise specified, the convolutional layer in the CNN model employs a kernel size of 2 × 2, a stride of 1, and a padding of 0. "GELU", an abbreviation for Gaussian Error Linear Unit activation function, introduces nonlinearity into the model architecture, enabling the model to learn complex patterns in the data.

## Appendix B A filing method for grid cell block

In this paper, a method of selectively filling the volume fractions of grid cells within the grid cell block column by column is adopted. This method first requires determining the filling direction of the grid cell block, which is based on the *n_y_*. The specific calculation method is shown in Eq. (24).

(24) $$n_{y}=\frac{\partial c}{\partial y}$$

If $n_{y}<0$, it indicates that the downward filling direction is adopted. The filling method for the volume fraction is shown in Eq. (25). The distribution of the filled volume fraction is illustrated in Fig. 28.

(25) $$\begin{array}{ccc} & & c_{i+d,j-e}\\ & & =\left\{ \begin{array}{c} c_{i+d,j-e},\text{if}c_{i+d,j-e}>c_{i+d,j-e+1}\text{and}\left|c_{i+d,j-e}-c_{i+d,j-e+1}\right|<\theta \\ 1.0,\text{if}c_{i+d,j-e}\leq c_{i+d,j-e+1}\text{and}\left|c_{i+d,j-e}-c_{i+d,j-e+1}\right|<\theta \end{array}\right.\left(\begin{array}{c} d=-2,-1,0,1,2\\ e=-1,0,1,2 \end{array}\right) \end{array}$$

If $n_{y}>0$, it indicates that the upward filling direction is adopted. The filling method for the volume fraction is shown in Eq. (26). The distribution of the filled volume fraction is illustrated in Fig. 29.

(26) $$\begin{array}{ccc} & & c_{i+d,j-e}\\ & & =\left\{ \begin{array}{c} c_{i+d,j-e},\text{if}c_{i+d,j-e}>c_{i+d,j-e-1}\text{and}\left|c_{i+d,j-e}-c_{i+d,j-e-1}\right|<\theta \\ 1.0,\text{if}c_{i+d,j-e}\leq c_{i+d,j-e-1}\text{and}\left|c_{i+d,j-e}-c_{i+d,j-e-1}\right|<\theta \end{array}\right.\left(\begin{array}{c} d=-2,-1,0,1,2\\ e=1,0,-1,-2 \end{array}\right) \end{array}$$

In this paper, the *θ* is set to 1.0 × 10^–3^.
